# Thermophilic *Dehalococcoidia* with unusual traits shed light on an unexpected past

**DOI:** 10.1038/s41396-023-01405-0

**Published:** 2023-04-11

**Authors:** Marike Palmer, Jonathan K. Covington, En-Min Zhou, Scott C. Thomas, Neeli Habib, Cale O. Seymour, Dengxun Lai, Juliet Johnston, Ameena Hashimi, Jian-Yu Jiao, Alise R. Muok, Lan Liu, Wen-Dong Xian, Xiao-Yang Zhi, Meng-Meng Li, Leslie P. Silva, Benjamin P. Bowen, Katherine Louie, Ariane Briegel, Jennifer Pett-Ridge, Peter K. Weber, Elitza I. Tocheva, Tanja Woyke, Trent R. Northen, Xavier Mayali, Wen-Jun Li, Brian P. Hedlund

**Affiliations:** 1 School of Life Sciences, University of Nevada Las Vegas, Las Vegas, NV 89154 USA; 2 State Key Laboratory of Biocontrol, Guangdong Provincial Key Laboratory of Plant Resources and Southern Marine Science and Engineering Guangdong Laboratory (Zhuhai), Sun Yat-Sen University, 510275 Guangzhou, People’s Republic of China; 3 Key Laboratory of Microbial Diversity in Southwest China of Ministry of Education, Yunnan Institute of Microbiology, School of Life Sciences, Yunnan University, 650091 Kunming, People’s Republic of China; 4 Department of Molecular Pathobiology, New York University College of Dentistry, New York, NY 10010 USA; 5 Department of Microbiology, Shaheed Benazir Bhutto Women University, Peshawar, Khyber Pakhtunkhwa (KPK) Pakistan; 6 Physical and Life Sciences Directorate, Lawrence Livermore National Laboratory, Livermore, CA USA; 7 Department of Microbiology and Immunology, Life Sciences Institute, The University of British Columbia, Vancouver, BC Canada; 8 Institute of Biology, Centre for Microbial Cell Biology, Leiden University, Leiden, The Netherlands; 9 The Department of Energy Joint Genome Institute, Berkeley, CA 94720 USA; 10 Environmental Genomics and Systems Biology Division, Lawrence Berkeley National Laboratory, Berkeley, CA 94720 USA; 11 Life and Environmental Sciences, University of California Merced, Merced, CA 95343 USA; 12 Innovative Genomics Institute, University of California Berkeley, Berkeley, CA 94720 USA; 13 Nevada Institute of Personalized Medicine, University of Nevada Las Vegas, Las Vegas, NV 89154 USA

**Keywords:** Phylogenetics, Bacteria, Metabolomics, Sequencing, Microbial ecology

## Abstract

Although the phylum *Chloroflexota* is ubiquitous, its biology and evolution are poorly understood due to limited cultivability. Here, we isolated two motile, thermophilic bacteria from hot spring sediments belonging to the genus *Tepidiforma* and class *Dehalococcoidia* within the phylum *Chloroflexota*. A combination of cryo-electron tomography, exometabolomics, and cultivation experiments using stable isotopes of carbon revealed three unusual traits: flagellar motility, a peptidoglycan-containing cell envelope, and heterotrophic activity on aromatics and plant-associated compounds. Outside of this genus, flagellar motility has not been observed in *Chloroflexota*, and peptidoglycan-containing cell envelopes have not been described in *Dehalococcoidia*. Although these traits are unusual among cultivated *Chloroflexota* and *Dehalococcoidia*, ancestral character state reconstructions showed flagellar motility and peptidoglycan-containing cell envelopes were ancestral within the *Dehalococcoidia*, and subsequently lost prior to a major adaptive radiation of *Dehalococcoidia* into marine environments. However, despite the predominantly vertical evolutionary histories of flagellar motility and peptidoglycan biosynthesis, the evolution of enzymes for degradation of aromatics and plant-associated compounds was predominantly horizontal and complex. Together, the presence of these unusual traits in *Dehalococcoidia* and their evolutionary histories raise new questions about the timing and selective forces driving their successful niche expansion into global oceans.

## Introduction

The phylum *Chloroflexota*, previously known as the “green non-sulfur bacteria” or *Chloroflexi*, is a diverse lineage in the Terrabacteria [1, 2], a major clade of bacteria whose ancestors likely colonized land early in Earth’s history [3]. Originally, the phylum consisted of a single class encompassing two orders of anoxygenic phototrophs and heterotrophs [4, 5], yet successful cultivation efforts have expanded the known breadth of the phylum, which now includes members with diverse physiological capacities, including respiration of oxygen, nitrate, ferric iron, and chlorinated organics; chemolithotrophic nitrite, carbon monoxide, or ferrous iron oxidation; fermentation; anoxygenic photosynthesis; and diazotrophy [5–10].

Despite successes cultivating diverse *Chloroflexota*, molecular surveys have shown that most *Chloroflexota* have not yet been cultivated. For example, the Genome Taxonomy Database (GTDB) [11] lists 12 classes and 73 orders, of which only four classes and eleven orders are represented by axenic cultures. These yet-uncultivated *Chloroflexota* lineages are abundant in a variety of biomes including marine [12–15], intertidal [14], and freshwater [14, 16, 17] environments, and extreme environments such as hypersaline mats [18], desert soil crusts [19], and geothermal springs [20]. An environmental meta-analysis showed *Chloroflexota* to be among the most abundant bacteria represented in both metagenomes [21] and metatranscriptomes from many biomes, including geothermal springs, the terrestrial subsurface, and bioreactors, with the metatranscriptomes suggesting high metabolic activity in situ [22]. Given the metabolic diversity, broad environmental distribution, abundance, and relative lack of axenic cultures of *Chloroflexota*, greater efforts are needed to better understand their physiology, ecology, and evolutionary history, particularly those belonging to lineages with few or no isolates.

One such lineage is the order *Tepidiformales*, previously described under the partially synonymous names GTDB order o_UBA2991, TK10, TK17, TK30, and OLB14. The first 16S rRNA gene sequences representing this order were recovered from marine sponges [23] where they are often part of the core microbiome [24]. Subsequently, additional sequences were retrieved from geothermal springs [25, 26], marine and freshwater environments, and soils. The lack of a cultured representative for this lineage prevented formal taxonomic description until recently, with the isolation of *Tepidiforma bonchosmolovskayae* from a geothermal spring as the sole cultivated member [27]. However, with only a single cultured representative, this group remains understudied, and its characteristics, diversity, and evolution largely unknown.

Here we report the isolation of two new species of *Tepidiforma* from hot springs, and provide insights into their physiology, ecology, and evolutionary history. Phylogenomic analyses placed this lineage as an order within the class *Dehalococcoidia*, and showed that it is widely distributed, with high prevalence in sediments and soils. A combination of cryo-electron tomography (cryo-ET), exometabolomics, and stable isotope probing coupled with cavity ring-down spectroscopy and nanometer-scale secondary ion mass spectrometry (nanoSIMS), revealed three unusual traits among members of the *Chloroflexota/Dehalococcoidia*, namely flagellar motility, a peptidoglycan-containing cell envelope, and metabolic activity on aromatics and plant-associated compounds, including recalcitrant organic matter. We then traced flagellar motility and peptidoglycan biosynthesis to the ancestor of the class and documented their loss, followed by expansion and diversification of the *Dehalococcoidia* in the global oceans.

## Results and discussion

### Two new *Tepidiforma* species expand on a small number of cultivated *Dehalococcoidia*

Two bacterial strains were isolated from terrestrial geothermal springs by plating benthic mat and sediment slurries onto R2A medium and were identified by 16S rRNA gene sequencing as members of the recently described genus *Tepidiforma* [27]. Strain YIM 72310^T^ was isolated from a microbial mat collected from Hamazui (Frog Mouth) Hot Spring in Rehai National Park, Tengchong, Yunnan Province, south-west China. Strain G233^T^ was isolated from sediment collected ~20 cm below the water level from the source pool of Great Boiling Spring (GBS), Nevada, USA.

**Fig. 1:**
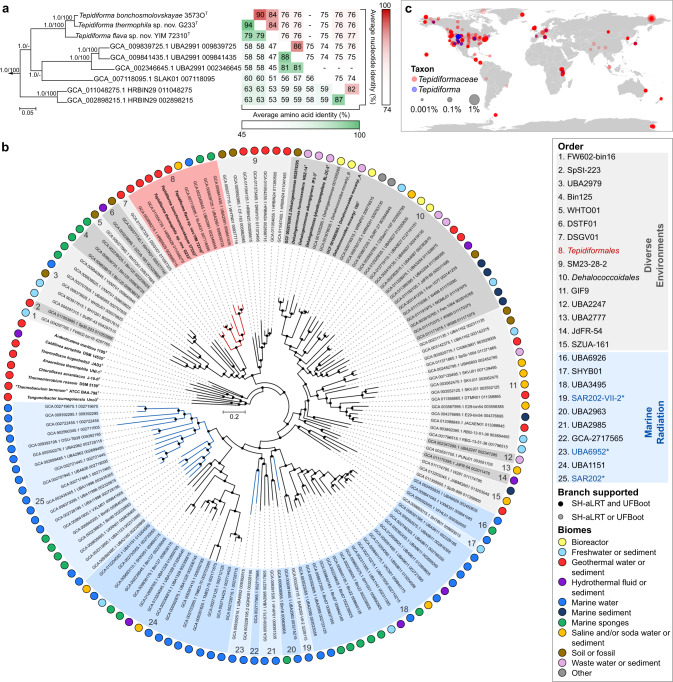
Phylogenetic placement and environmental distribution of *Tepidiforma* in the class *Dehalococcoidia*. **a** Subtree of the order *Tepidiformales*, extracted from the maximum-likelihood phylogeny for the *Dehalococcoidia* (**b**), with overall genome relatedness indices, Average Nucleotide Identity (ANI) and Average Amino Acid Identity (AAI), indicated for the two novel *Tepidiforma* species, *T. thermophila* and *T. flava* compared to other members of the order. Branch support was inferred from 1000 replicates each using Shimodaira-Hasegawa approximate Likelihood Ratio Test (SH-aLRT) and ultrafast bootstrap (UFBoot) support and is indicated at nodes as (SH-aLRT/UFBoot). The scale bar indicates the number of amino acid changes per site. **b** Maximum-likelihood phylogeny for the class *Dehalococcoidia*, constructed from a concatenation of 120 conserved bacterial markers (Bac120 dataset), with partitioning per marker and application of independent evolutionary models per partition. Each taxon represents a single species-level group, using the highest quality genome as representative where cultivated representatives are lacking, and cultivated members of the *Chloroflexota* from other classes were included as outgroups. All genomes derived from isolates are indicated in bold (nine in *Dehalococcoidia*). The scale bar indicates the number of amino acid changes per site, and supported branches are indicated with dots at nodes (support from 1000 replicates using SH-aLRT and UFBoot). GenBank assembly accession numbers and GTDB associated taxon identifiers are indicated for each taxon included in the analysis. The order *Tepidiformales* (**a**) is shaded in red and indicated with red branches, while other orders from diverse environments within the class are shaded in gray, and the Marine Radiation is shaded in blue. Confidently identified SAR202 lineages are labeled in blue with an asterisk in the key and indicated with blue branches. Biomes from which genomes were recovered, or species were isolated from, are indicated by colored dots in the outer ring. **c** Global environmental distribution of the family *Tepidiformaceae* and genus *Tepidiforma* based on amplicon sequence variants from the Earth Microbiome Project.

Genomes of all *Tepidiforma* species ranged between 2.74 and 2.77 Mbp (see Supplementary Note 1) and enabled identification of the new isolates as novel species in the genus *Tepidiforma*, with ANI values well below suggested species delineation guidelines [28]. These new species are herein proposed as *Tepidiforma flava* YIM 72310^T^ and *Tepidiforma thermophila* G233^T^ (Fig. 1). To address the phylogenetic and taxonomic position of the genus, a large-scale phylogenomic analysis of the *Chloroflexota* was performed, leveraging the GTDB release 202 [11], which contains 694 high-quality *Chloroflexota* genomes, with the majority (>650) represented by metagenome-assembled genomes (MAGs) from uncultivated taxa. Of these, 392 high-quality species representatives were included in our phylogenomic reconstruction (Fig. S1 and Table S1), which used 120 conserved marker proteins that were concatenated, partitioned, and analyzed under individual evolutionary models, using maximum likelihood. This analysis resulted in a robust, well-supported phylogeny for the phylum (Fig. S1). Further discussion of the phylogenetic and taxonomic structure of the *Chloroflexota* is included in Supplementary Note 2. The two new species, *T. bonchosmolovskayae*, and 148 additional high-quality genomes (nine derived from isolates) were placed within the class *Dehalococcoidia* (Fig. 1b and Table S2). Together, these data support the designation of this lineage as the order *Tepidiformales*, containing a single family, *Tepidiformaceae*, in the class *Dehalococcoidia* (Fig. 1a, b).

### Environmental distribution and phylogenomics of *Dehalococcoidia* supports transitions into saline and marine habitats

Despite the class *Dehalococcoidia* being largely associated with marine environments [1, 29, 30], biome data associated with *Dehalococcoidia* genomes indicate a wide environmental distribution for this class and the order *Tepidiformales* (Fig. 1b). MAGs splitting from basal nodes (orders 1–15) are derived from diverse environments, consistent with a terrestrial origin of the Terrabacteria and phylum *Chloroflexota*, while shallow-branching taxa (orders 16–25) are almost exclusively from marine environments (Fig. 1b). This evolutionary pattern is mirrored in the order *Tepidiformales*, where taxa splitting at basal nodes are thermophilic isolates or MAGs from terrestrial thermal environments, and genomes at shallow-branching nodes (genera SLAK01 and UBA2991) were recovered from a hypersaline soda lake, marine water, or sponges (Fig. 1b).

To further interrogate the distribution of this lineage, we identified amplicon sequence variants (ASVs) from the Earth Microbiome Project (EMP) (see Supplementary Note 3). SILVA lineages OLB14 and TK30 were retained, while the neighboring lineages TK17, TK10, and N9D0 were excluded as they could not confidently be mapped to the family or order (Table S3). This analysis revealed insights into the geographic distribution of the *Tepidiformaceae* and their presence in different biomes, as defined through three levels within the EMP ontology (empo) (for explanation of the EMP database and empo categories, see Supplementary Note 3 and [31]). Based on EMP data, the *Tepidiformaceae* are globally distributed (Table S3 and S4), with the highest prevalence in the empo-2 categories non-saline (30%) (Fig. 1c), plant (22%), and saline (16%), albeit at low relative abundance (<1%) (Fig. S2 and Table S3, S4). At the level empo-3, the family was most prevalent in the plant rhizosphere (89%), followed by soils (65%), saline sediments (32%), and non-saline sediments (31%). However, the biome coverage of the EMP is limited, so this inventory should be treated as preliminary. For example, although *Tepidiformaceae* are prevalent in marine sponges (Fig. 1b), sponges are not included in the EMP animal microbiome dataset. Most members of the genus *Tepidiforma* were detected in alkaline pH soils or waters (8.65–9.22) or terrestrial geothermal springs with temperatures similar to the growth range for *Tepidiforma* isolates (35.8–68 °C) (Fig. S2 and Table S3, S4).

Our analyses provide strong evidence for the presence of older lineages in *Dehalococcoidia* (orders 1–15), including *Tepidiformales* (order 8), in diverse environments, with a major one-way radiation into the oceans (“Marine Radiation”, orders 16–25, including pelagic, sponge, and hydrothermal vent communities). Our interpretations contradict a recent analysis of 16S rRNA gene sequences from *Dehalococcoidia* genomes [32], which proposed an earlier divergence of marine cluster 1, and subsequent divergence between marine cluster 2 (including the orders JdFR-54 [order 14 in Fig. 1] and GIF9 [order 11 in Fig. 1]), and a terrestrial cluster consisting of members of the *Dehalococcoidales* (order 10 in Fig. 1b). However, these marine “clusters” were not consistently monophyletic in 16S rRNA gene phylogenetic reconstructions [32] and they were not recovered in our genome-based analyses, as high-quality representatives of groups included in marine cluster 2 were not monophyletic (and not solely marine-associated), and representatives of marine cluster 1 were of too low quality to be included in our analyses. The results from this study do align with a previous hypothesis of a freshwater origin of major groups of pelagic *Dehalococcoidia* and their subsequent transition to saline/marine environments [17]. This interpretation is consistent with the predisposition of marine *Dehalococcoidia*, including the SAR202 lineage, to accumulate genes for the degradation of recalcitrant organic matter of terrestrial origin [15, 33] (see below), an unusual genomic remnant of a terrestrial ancestry.

### Flagellar motility and cell ultrastructure are unique among known *Chloroflexota* and *Dehalococcoidia*

**Fig. 2:**
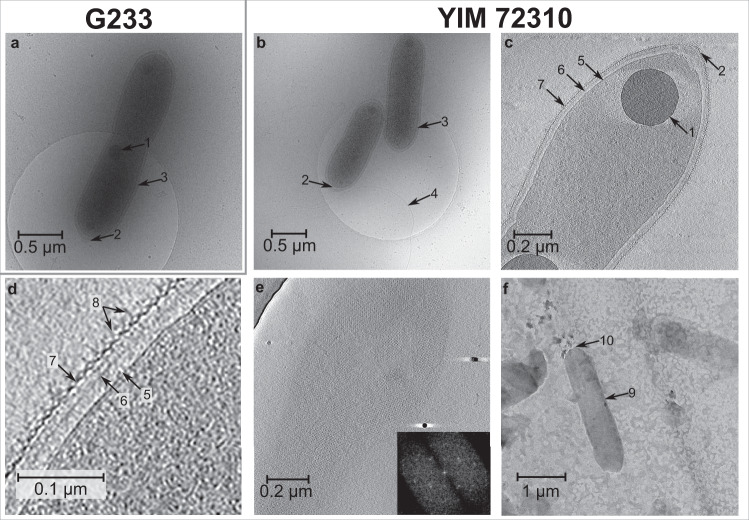
Cryo-electron tomography of the strains G233^T^ (*T. thermophila*) and YIM 72310^T^ (*T. flava*). Cells of strains G233^T^ (**a**) and YIM 72310^T^ (**b**) are rod-shaped, with polyphosphate granules (1) and conical cell poles (2). **a**–**d** The cell envelope (3) consists of the cytoplasmic membrane (5), a peptidoglycan layer (6), and an S-layer (7) with unusual protein complexes (8). A flagellum (4) is visible for YIM 72310^T^ (**b**). **e** Uniform tetragonal lattice of the S-layer of YIM 72310^T^. **f** Sacculus (9) of strain YIM 72310^T^ formed by cell lysis and enzymatic digestion with trypsin with rounded cell poles (10).

Phenotypic experiments and cryo-ET (Fig. 2) of the novel *Tepidiforma* species revealed traits that are unusual among cultivated *Chloroflexota* and *Dehalococcoidia*, including flagellar motility and cell envelopes containing peptidoglycan. Flagellar motility was observed in cultures of both novel isolates of *Tepidiforma* and reported previously for *T. bonchosmolovskayae*. Similarly, 2-dimensional cryo-EM images revealed a flagellum for strain YIM 72310^T^ (Fig. 2b). These *Tepidiforma* isolates are the only members of the *Chloroflexota* known to possess flagella. Many cultivated members of the *Chloroflexota* are considered non-motile [7, 34, 35], although gliding motility in this phylum is well known, particularly in the filamentous *Chloroflexia* [9, 36–39]. Despite these observations based on isolates of *Chloroflexota*, environmental genomics studies have improved representation of the diversity across the phylum and revealed flagellar gene clusters in some *Chloroflexota* genomes [17, 36, 40–42]. However, direct evidence for flagella in members of the phylum is so far limited to the genus *Tepidiforma* (Fig. 2b and noted previously in [27]).

Cryo-tomograms of the *Tepidiforma* strains also revealed, from in to out, a 7 nm cytoplasmic membrane, a 40 nm periplasm containing a ~20 nm layer suggestive of peptidoglycan, and an S-layer (Fig. 2c and d) (see Supplementary Note 4 for further description), consistent with transmission electron micrographs (TEMs) of *T. bonchosmolovskayae* cells [27]. Here, we present direct evidence that the cell envelopes of the new *Tepidiforma* strains do indeed contain peptidoglycan: (i) TEMs of purified sacculi [43] formed by cell lysis and enzymatic digestion with trypsin (Fig. 2f); (ii) inhibition of growth by β-lactam antibiotics (Fig. S3); and (iii) a cell-wall hydrolysate containing threonine, alanine, and proline as major amino acids, and glycine and valine as minor amino acids. However, no diaminopimelic acid (DAP) or lysine was identified from the hydrolysate. This unusual finding coupled with the annotation of the DAP-type peptidoglycan biosynthesis pathway may suggest the use of modified amino acids in their cell envelopes, justifying further inquiry.

Cryo-ET also revealed other structural features that are distinct from known organohalide-respiring *Dehalococcoidia*. Members of the order *Dehalococcoidales* range in cell shape from irregular discs (*Dehalococcoides mccartyi* [44, 45]) to irregular or slightly flattened cocci (*Dehalogenimonas* spp [6, 45, 46]). In contrast, cells of *Tepidiforma* are rod-shaped and range in size between 1.0 and 2.5 µm long by 0.2–0.4 µm wide and contain unusual cone-like structures at cell apices, similar to cells of *T. bonchosmolovskayae*. Sacculi lacked these cone-like structures, confirming they are likely formed by the S-layer and not peptidoglycan (Fig. 2f). The S-layer surrounding the cells was uniform and 2D Fourier transforms revealed a tetragonal lattice (Fig. 2e). This is similar to some cultivated *Dehalococcoidaceae* species [45], but unlike other *Chloroflexota* where hexagonal S-layers only cover cell apices [39].

Unusual protein complexes, spaced at ~9 nm, were observed on both sides of the S-layer (Fig. 2d). As several members of the class *Dehalococcoidia* respire organohalides [6, 32, 36, 44, 47–49], we considered the possibility that these protein complexes may represent a dehalogenation apparatus. However, no distinct densities were observed associated with the cytoplasmic membrane, and, like other members of this class outside of the *Dehalococcoidales*, the *Tepidiforma* genomes had no identifiable homologs of any components of the reductive dehalogenase protein complexes included in the Reductive Dehalogenase Database (RDaseDB [48]).

Both species also had intracellular polyphosphate granules at cell division planes that were ~200 nm in diameter (Fig. 2 and [27]) and genes involved in polyphosphate synthesis and degradation [50, 51] were present in all *Tepidiforma* genomes (see Supplementary Note 4 for details). Polyphosphate plays an important role in oligotrophic environments as an energy storage compound [16, 50, 51], regulates cell cycle exit during starvation [51], and is also known to be important for flagellar motility [50], possibly playing a role in synthesis, stability, or function of the flagellar motor [52].

Overall, the unusual traits of *Tepidiforma*, especially flagellar motility and peptidoglycan-containing cell envelopes, are unexpected for members of the phylum and class, respectively. For this reason, the phylogenetic distribution and evolutionary history of these traits, along with the metabolic capacity of the new *Tepidiforma* species, were further investigated.

### Molecular remnants of flagellar motility are ancestral in *Dehalococcoidia* and pervasive in the *Chloroflexota*

**Fig. 3:**
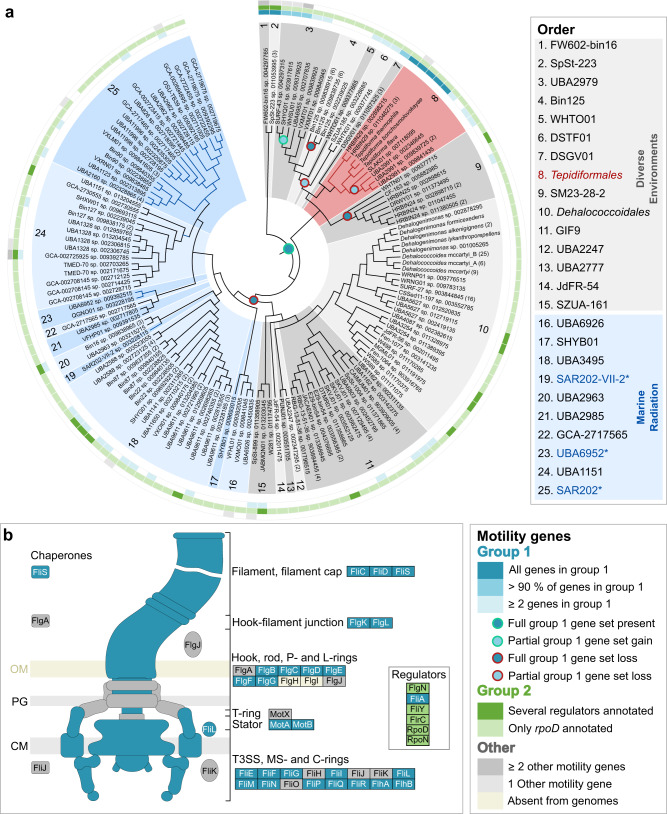
Distribution and evolution of motility within the class *Dehalococcoidia*. **a** Cladogram inferred from the *Dehalococcoidia* phylogenomic tree serving as evolutionary hypothesis for ancestral character state reconstruction. Orders from diverse environments are shaded in gray, while the *Tepidiformales* are shaded in red and indicated with red branches, and the Marine Radiation is shaded in blue, with SAR202 lineages indicated with blue branches and asterisks and blue text in the key. The presence of a core structural flagellar gene set (Group 1 genes) in genomes belonging to the class is indicated with gradations of teal in the inner track, while the presence of regulatory genes (Group 2 genes) is indicated in gradations of green in the middle track. Other motility genes that are intermittently found within the class are indicated in gray in the outer track. Predicted consensus gains (dots outlined with green) or losses (dots outlined with red) of the full or partial core structural set are indicated at nodes where predictions for individual genes agree. **b** Schematic of conserved flagellar genes found in the genomes of the *Dehalococcoidia*. Conserved structural and regulatory proteins are indicated based on their relative positions on the flagellum schematic while those components found intermittently are indicated in gray, and components being absent from the genomes are indicated in light yellow, specifically FlgH and FlgI, forming the L- and P-rings.

To evaluate the distribution of flagellar genes in the *Dehalococcoidia*, all genomes for the class included in this study were compared. Genes encoding flagellar machinery were grouped (Fig. 3) into a core set of often co-occurring genes (teal-colored genes), regulatory genes (green-colored genes), and other genes that were intermittently present (gray-colored genes). All genomes (243) representing 140 species clusters, except for the sole high-quality representative of GCA_009391415.1 sp. 009392785, lacked the *flgH* and *flgI* genes that encode L-and P-rings, consistent with the monoderm Gram-positive cell envelope structure of *Chloroflexota*. The core gene set (>90% of the 25 genes) was frequently observed in the taxa splitting off at the basal nodes within the class (orders 1, 2, 3, 7), but individual genes were rarely present in other members of the class (Fig. 3a).

From the observed distribution patterns, we reconstructed likely ancestral character states for these genes. The core flagellar gene set was predicted in the last common ancestor of the class, with a few partial or full gene losses in taxa branching at basal nodes (i.e., ancestors of orders 3–6 and 9, and within *Tepidiformales*), and a full loss of the cluster in the last common ancestor of orders 10–25, prior to the diversification of the lineages including the *Dehalococcoidales* and the Marine Radiation comprising orders 16–25 (Fig. 3a). Within the *Tepidiformales* and its sister order DSGV01, the core flagellar gene set was present in all genomes retrieved from geothermal environments, with partial losses of flagellar genes predicted at ecological transitions to cooler environments (e.g., genera SLAK01 and UBA2991).

As *Tepidiforma* is aerobic or microaerophilic and often associated with phototrophic mats, it is possible that flagellar motility could be selected for by changes in conditions during redox cycling in photosynthetic mats. Diel vertical migration via gliding motility of *Chloroflexus aurantiacus* [53] and other taxa within chlorophototrophic mats has been a topic of interest for nearly half a century [53]. In these settings, aerobes typically accumulate in the uppermost layers of mats at night [53], as lower layers become anoxic [54, 55]. In contrast, O_2_ supersaturation occurs within photosynthetic layers during high light, leading to accumulation of reactive oxygen species (ROS) [54], driving many microorganisms deeper into mats and sediments. All three members of the *Tepidiforma* could also potentially alleviate ROS-induced damage by using glutathione peroxidase, which is conserved in *Tepidiforma*, and superoxide dismutase, which is conserved in the *Tepidiformales* (Table S5).

**Fig. 4:**
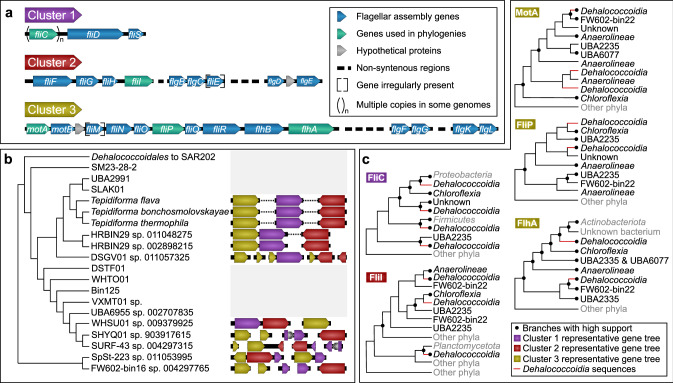
Gene cluster architecture and evolutionary hypotheses for motility within the *Dehalococcoidia*. **a** Three non-contiguous gene clusters encoding motility genes were identified in *Dehalococcoidia* genomes. Both clusters 2 and 3 contained non-syntenic regions nested within the cluster, while *fliE* (cluster 2) and *fliM* (cluster 3) was irregularly present, and some genomes encoded multiple copies of *fliC* (cluster 1). **b** The genomic organization of gene clusters within *Dehalococcoidia* genomes, plotted against the evolutionary relatedness of the class as inferred from the phylogenomic tree. The branch leading to the ancestor of the *Dehalococcoidales* and the Marine Radiation is collapsed on the cladogram, as none of these genomes contained the consistent gene clusters observed in predicted motile *Dehalococcoidia*. **c** Summary cladograms for individual maximum-likelihood trees (Supplementary Data file 1) for representative sequences from each cluster (genes indicated in green in **a**). Branches supported with both SH-aLRT (>0.9) and UFBoot (>95%) are indicated with filled dots at nodes. Lineages belonging to the *Dehalococcoidia* are indicated with red branches. Lineages classified as belonging to classes in the *Chloroflexota* based on GTDB are reflected in black taxon names, and those lineages belonging to phyla outside of the *Chloroflexota* are indicated in gray taxon names.

Investigation of the genomic architecture and synteny of the flagellar genes revealed three gene clusters (Fig. 4). Cluster 1 (purple) consisted of genes associated with the flagellar filament and filament cap (*fliC*, *fliD*, and *fliS*); Cluster 2 (red) consisted of some *fli* and *flg* genes; while Cluster 3 (gold) contained the remainder of the *fli* and *flg* genes, along with *mot* and *flh* genes (Fig. 4a). Most of the more fragmented genomes could be shuffled to reflect a similar structure (Fig. 4b), except for SpSt-233 sp. 011053995, where assembled contigs did not reflect this organization, and the sister taxa SHYQ01 sp. 903917615 and SURF-43 sp. 004297315, which each contained an insertion in Cluster 1 (Fig. 4b).

To gain insight into the evolutionary history of the flagellar genes within the class, proteins encoded by the different flagellar gene clusters were selected for phylogenetic analyses: flagellin FliC (Cluster 1), the flagellum-specific ATP synthase FliI (Cluster 2), the motor complex protein MotA, and the flagellar biosynthesis proteins FliP and FlhA (Cluster 3) (see Supplementary Note 5 for more detail). Phylogenetic analyses showed that *Dehalococcoidia* homologs consistently grouped with other members of the *Chloroflexota*, particularly in the classes *Anaerolineae*, *Chloroflexia*, FW602-bin22, UBA2235, and UBA6077. When other phyla grouped close to sequences from the *Chloroflexota*, these mostly belonged to other Terrabacteria [2], such as *Actinobacteriota*, *Armatimonadota*, *Cyanobacteria*, *Eremiobacterota*, and *Firmicutes* (syn. *Bacillota*). There were, however, some examples of putative horizontal transfer events between some Gracilicutes or Terrabacteria and *Dehalococcoidia*. For example, some FliC copies from *Dehalococcoidia* grouped closest to *Proteobacteria* sequences (syn. *Pseudomonadota*), while one lineage of FliI sequences from *Dehalococcoidia* grouped closest to sequences from the *Planctomycetota* (Fig. 4c). However, the widespread presence of these flagellar genes within the *Chloroflexota* suggests that flagellar motility may have been ancestral within the phylum, and was mainly inherited vertically with several losses, homologous replacements, and duplications occurring within the phylum. Overall, these phylogenies reflect a complex evolutionary history for flagellar genes in the *Chloroflexota* that warrants further investigation but may also reflect the limitations to robust reconstruction of evolutionary paths of single genes in deep time.

### Peptidoglycan biosynthesis is an ancestral trait in the *Dehalococcoidia*

A layer suggestive of peptidoglycan, ranging between 20 and 30 nm in thickness, was visible by electron microscopy in all three cultivated *Tepidiforma* species, and was herein confirmed as peptidoglycan. The non-thermophilic *Dehalococcoidia* genera *Dehalococcoides* and *Dehalogenimonas* have either been reported to lack peptidoglycan in their cell envelopes [44, 45, 56], or no data are available regarding envelope architecture [6, 46, 47]. The apparent lack of peptidoglycan was previously also supported by the available genome data, as evidence for peptidoglycan biosynthesis has been absent from published genomes [1, 36, 57]. In addition, all *Tepidiformales* genomes lacked key genes for the synthesis of a Gram-negative outer membrane, supporting their designation as monoderms, matching the current paradigm for *Chloroflexota* genomes [40], and other members of the Terrabacteria [58].

**Fig. 5:**
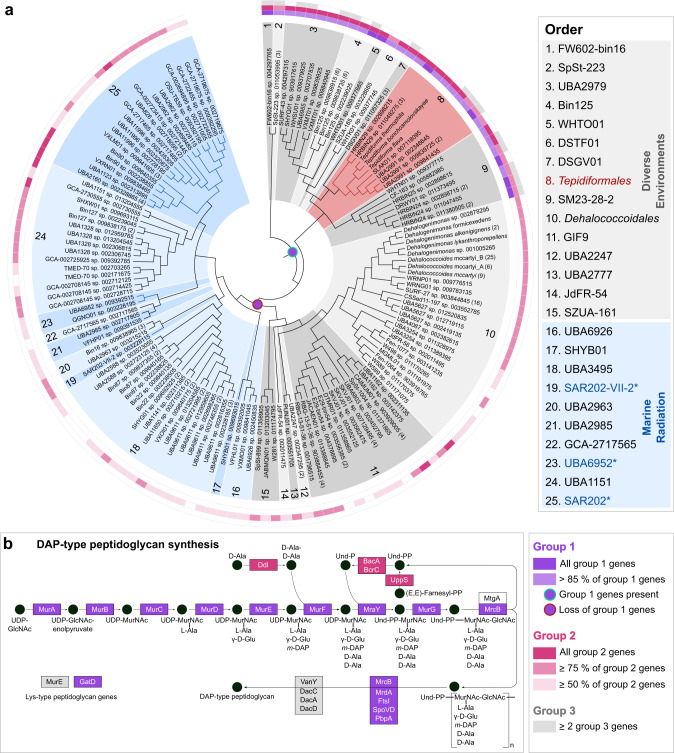
Distribution and evolution of peptidoglycan biosynthesis within the class. **a** Cladogram inferred from the *Dehalococcoidia* phylogenomic tree serving as evolutionary hypothesis for ancestral character state reconstruction. Orders from diverse environments are shaded in gray, while the *Tepidiformales* are shaded in red and indicated with red branches, and the Marine Radiation is shaded in blue, with SAR202 lineages indicated with blue branches and asterisks and blue text in the key. The presence of three sets of conserved genes is indicated on the cladogram. Group 1 genes encode proteins for the central DAP-type peptidoglycan biosynthetic pathway (purple), Group 2 (pink), and Group 3 (gray) genes encode proteins involved in peptidoglycan synthesis and other metabolic pathways for amino acids and secondary metabolites. The full Group 1 gene set is predicted as present in the ancestor to the class (purple dot with green outline), with a full loss of Group 1 genes predicted at the node reflecting an ancestor to orders 10–25. **b** Diagram of the DAP-type peptidoglycan biosynthetic pathway, and the relative position of enzymes encoded by the different gene sets within the pathway.

To probe the likely cell envelope structure across *Dehalococcoidia*, we interrogated the genomes for peptidoglycan biosynthetic potential. Nearly all genes associated with the DAP-type peptidoglycan biosynthesis pathway were present in orders 1–9 (Fig. 5, purple genes), including the *Tepidiformales* (order 8). In addition, the genes *ddl*, *bacA*, *bcrC*, and *uppS* were widely present within the class (Fig. 5a), suggesting DAP-type peptidoglycan biosynthesis might be ancestral within the class. This hypothesis was supported by ancestral character state reconstructions (see Supplementary Note 5), which showed a single loss of the core DAP-type peptidoglycan biosynthesis genes at the last common ancestor of orders 10–25 (Fig. 5a; including the *Dehalococcoidales* and the Marine Radiation)—the same node at which flagellar motility was lost.

### *Tepidiforma* isolates exhibit broad heterotrophic activity, including degradation of aromatics

Physiologies within the *Dehalococcoidia*, both known and predicted, differ markedly. Though cultivated members of the class are best known for reductive dehalogenation [6, 44–48] or consortial degradation of recalcitrant organic carbon, syntrophic interactions have been suggested based on MAGs [13, 32, 49] and known reductive dechlorination reactions [59]. For example, some marine *Dehalococcoidia* that encode genes involved in the degradation of aromatic and plant-derived organic compounds have been predicted to partially degrade terrestrial recalcitrant organic carbon (e.g., alicyclic compounds [15]), where the products can then be used by other community members [15, 33, 57, 60, 61].

**Fig. 6:**
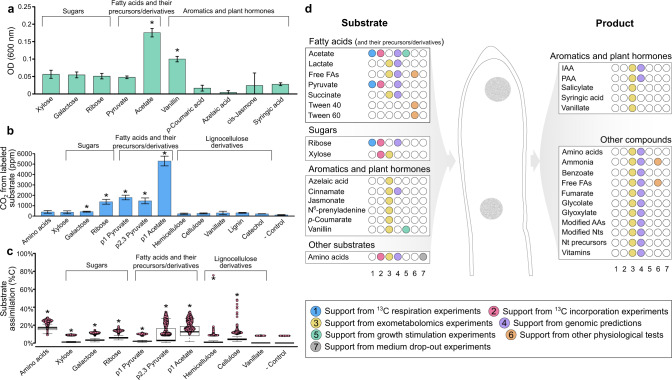
Metabolism of strain YIM 72310^T^ as determined from physiological tests, isotopically labeled ^13^CO_2_ production and assimilation assays, exometabolomics and genomic predictions. **a** Growth stimulation tests performed through addition of carbon sources to 2R2AW at a concentration of 0.05% w/v, after 8 days of growth as measured by optical density at 600 nm. Statistically significant stimulation (indicated with an asterisk) was determined based on unpaired *t*-tests (*p* < 0.05) compared to controls for the respective experiments. **b** Average relative ^13^CO_2_ production from growth on ^13^C-labeled substrates (triplicate incubations ± standard deviations). Statistical significance (indicated with asterisks) was determined through comparison to the negative control, with no ^13^C-labeled substrates, with Dunn tests. **c** Assimilation of ^13^C-labeled substrates was calculated from nanoSIMS analyses of individual cells (see Supplementary Note S7). Substrate biomass assimilation of individual cells, shown in each data point was calculated using C_net_ and plotted with a boxplot overlay for the average percent incorporation and the upper and lower quartile ranges. Significant assimilation of ^13^C (indicated with asterisks) was determined through comparison to the negative control with Dunn tests. **d** Dot matrix summarizing the metabolic capabilities of YIM 72310^T^. Substrates and products identified through exometabolomics are indicated with yellow dots, while supporting evidence based on ^13^CO_2_ production (blue dots) and assimilation (pink dots) experiments, genomic predictions (purple dots) and growth tests (green, orange, and gray dots) are also indicated. P1 Pyruvate, first carbon atom is labeled with ^13^C; P2,3 Pyruvate, second and third carbon atoms are labeled with ^13^C, FAs fatty acids, IAA indole-3-acetic acid, PAA phenylacetic acid, AAs amino acids, Nts nucleotides, Nt nucleotide.

To determine whether the new *Tepidiforma* species play a role in the metabolism of recalcitrant organic matter and improve our general understanding of the metabolic capacity of *Tepidiforma*, routine physiological characterization and omics-informed growth experiments were coupled with exometabolomics and targeted stable isotope experiments. Both strains only grew chemoorganotrophically under oxic conditions (Table S8), and their genomes encoded no known homologs of genes for autotrophy, phototrophy, or use of alternative terminal electron acceptors, including organohalides (Table S6–S8; see Supplementary Note 6 for details), unlike the organohalide-respiring *Dehalococcoidales*, and many anaerobic marine *Dehalococcoidia* that encode the Wood-Ljungdahl pathway [1, 29]. In contrast, the closest relative, *T. bonchosmolovskayae* was described as a facultative chemolithoautotroph capable of using siderite (FeCO_3_) as the electron donor, yet no carbon fixation pathway was proposed [27]. In addition, unlike *T. bonchosmolovskayae* [27], neither novel strain was capable of growth on any of the compounds tested as sole carbon sources (Table S9, S10, S11, and Supplementary Note 6), but several stimulated growth (Table S11) when added to undefined nutrient-rich media (2R2AW broth), which could indicate metabolic dependencies on other community members in situ. As such, two additional experiments were conducted to probe the metabolism of organic molecules: (i) exometabolomics experiments for both strains grown in R2A broth, where extracellular metabolites produced or consumed were detected with strict metabolite identification standards and statistical filtering (Fig. S4, Table S12–17); (ii) growth in 2R2AW broth with added ^13^C-labeled substrates followed by measurements of mineralization of organic substrate to ^13^CO_2_ and intracellular incorporation using isotope imaging (Figs. 6, S5, and S6). The ^13^C-labeled substrates tested (i.e., amino acids, xylose, galactose, ribose, pyruvate, acetate, vanillate, lignin, hemicellulose, cellulose, and catechol) in the latter experiments were chosen based on genome-enabled predictions or by exometabolomics experiments.

Based on growth (Fig. 6a and d, Table S10–11, Supplementary Note 6), exometabolomics (Fig. 6d, S4, and Table S12–13), and experiments with ^13^C-labeled substrates (Fig. 6b, c, and d, and Fig. S5), volatile fatty acids and their precursors or derivatives were consistently used for growth and/or energy generation. This was particularly evident for acetate, which stimulated growth (Fig. 6a and Table S11) and was both oxidized to ^13^CO_2_ (Fig. 6b and S5) and incorporated (Fig. 6c) (see Supplementary Note 7). The lack of a complete glyoxylate cycle encoded by *Tepidiforma* genomes (Table S6–7 and [27]) is consistent with their inability to use acetate as a sole carbon source (Table S9), and suggests its stimulatory effect on growth could be due to the production of acetyl-CoA or incorporation into membrane fatty acids. With this in mind, *Tepidiforma* could utilize acetate released by cohabiting acetogens in nature, similar to the well-documented utilization of acetate among other *Chloroflexota* [62, 63]. Although pyruvate did not stimulate growth, ^13^CO_2_ was produced and ^13^C was incorporated from both ^13^C_1_-pyruvate and ^13^C_2,3_-pyruvate (Fig. 6), and pyruvate was reported to support heterotrophic growth by *T. bonchosmolovskayae* [27]. The high levels of ^13^C_1_-pyruvate converted to ^13^C-CO_2_ were consistent with strong pyruvate dehydrogenase activity and tight coupling of glycolysis and the tricarboxylic acid cycle. Although neither succinate nor propionate stimulated growth of either strain, succinate served as substrate for both strains in exometabolomics experiments (Fig. 6d, Table S12–13), providing some evidence for cross-feeding interactions similar to those reported previously for *Roseiflexus* in hot spring microbial mats [55, 64]. Furthermore, exometabolomics demonstrated metabolism of lactate and other volatile fatty acids during growth on R2A, and the formation of halos around colonies grown on solid R2A medium amended with polysorbate [65] indicated that both strains could hydrolyze polysorbate (Tween) 40 and 60. Together, these data suggest that, like other *Chloroflexota*, fatty acids and their precursors and/or derivatives play an important role in sustaining growth of *Tepidiforma* species [27].

Although no sugars stimulated growth, other experiments showed that several sugars are utilized. Based on genomic data, we predicted ribose could be transported and used for nucleotide biosynthesis via the non-oxidative pentose phosphate pathway and indeed label from ^13^C_1_-ribose was recovered as ^13^CO_2_ (Fig. 6b) and in biomass (Fig. 6c). In addition, xylose was consumed during exometabolomics experiments by both strains, while only small amounts of ^13^C incorporation from ^13^C-xylose was detected by nanoSIMS. Some limited and sporadic evidence for the utilization of other sugar mono- or dimers was detected from the different experiments (e.g., ^13^CO_2_ production and ^13^C incorporation during growth in the presence of ^13^C-galactose Fig. 6c), however, these results could not be corroborated by other growth experiments or through genomic predictions. Several glycoside hydrolases (GHs) were predicted for both YIM 72310^T^ and G233^T^ (Table S18–19), particularly belonging to GH family 13, which contains various enzymes including amylases and pullulanases known to depolymerize starch, glycogen, and other polysaccharides; however, none of the polysaccharides tested (i.e., dextrin, starch, and polysaccharides in yeast extract) could be used as sole carbon sources, stimulated growth, or reduced growth when excluded from the medium (Tables S9–11). Yet, some ^13^C from both hemicellulose and cellulose were incorporated into biomass, suggesting a role for *Tepidiforma* in consortial degradation of polysaccharides, consistent with the growth of *T. bonchosmolovskayae* on plant polysaccharides such as starch and dextrin [27], and similar to other cellulolytic *Chloroflexota* [5]. Yet, since these hemicellulose and cellulose preparations are derived from plant matter, the exact source of the assimilated carbon atoms could be further investigated.

Plant biomass also contains aromatic compounds, including those that are integral to the structure of lignin. Broad metabolism of phenolics and other aromatic compounds was observed with both growth stimulation and exometabolomics experiments (Tables S11–13) (Fig. 6). However, despite the apparent use of aromatics by both strains during exometabolomic experiments, some related compounds accumulated during growth. For example, hippuric acid may be hydrolyzed by hippurate hydrolase (EC 3.5.1.32) to benzoic acid, while cinnamate is likely converted to benzoic acid and acetate through β-oxidation, with further processing of benzoic acid to salicylate through benzoate oxidoreductase. Similarly, *p*-coumaric acid may undergo β-oxidation to produce 4-hydroxybenzoic acid, while 3-hydroxybenzyl alcohol may be dehydrogenated to 3-hydroxybenzaldehyde, and further processed to 3-hydroxybenzoic acid through 3-hydroxybenzyl-alcohol dehydrogenase (EC 1.1.1.97) and benzaldehyde dehydrogenase (EC 1.2.1.28). Thus, these substrates are only partially or inefficiently metabolized, pointing to a possible role in consortial degradation of lignin or other recalcitrant organic matter [15] of plant and/or microbial origin in geothermal springs. However, vanillin stimulated growth, apparently via dehydrogenation to vanillate (inferred from exometabolomics) and subsequent mineralization, although not statistically significant, providing more compelling evidence for a direct role in lignin metabolism, particularly as growth on lignin was reported for *T. bonchosmolovskayae* [27]. Our growth experiments on ^13^C-lignin, catechol, and vanillate indicated mineralization of these compounds (Figs. S6, S5, and S6), but no intracellular incorporation was detected, though lignin and catechol adhered to the cell surface (Fig. S5), confounding nanoSIMS experiments (see Supplementary Note 7). These results are broadly consistent with the consortial nature of efficient lignin biodegradation in general [66], although high temperatures in geothermal systems might also alleviate enzymatic, kinetic, or thermodynamic bottlenecks for some of these reactions in situ [67]. Although limited genomic evidence is available to support these reactions, possibly due to difficulty in annotating proteins of novel organisms, the confirmed broad activity on these compounds is consistent with the proposed metabolism of aromatic compounds by yet-uncultivated marine *Dehalococcoidia*, specifically the SAR202 cluster (Fig. 1b, including orders 18 [partially synonymous with SAR202-III/*Monstramariales*], 19 [syn. SAR202-VII], 23 [syn. SAR202-VI], and 25 [syn. SAR202-II], indicated in blue and an asterisk in the key) [15, 33] and the Dsr clade [33, 57]; however, the distributions and phylogenies of enzymes associated with these degradation pathways are complex, with horizontal acquisition of these genes being pervasive (Fig. S7, S8, and Supplementary Note 5).

Plant stress hormones (i.e., azelaic acid and jasmonic acid) also served as substrates for both strains in exometabolomics experiments, while the plant growth hormones indole-3-acetic acid and phenylacetic acid (i.e., auxins) were produced (Table S12–13). Although not supported by genomic evidence, indole-3-acetic acid may be produced from 3-(2-methylaminoethyl)indole (a substrate consumed during exometabolomics experiments) using activities similar to monoamine oxidase (EC 1.4.3.4), and indole-3-pyruvic acid using activities similar to indole-3-pyruvate monooxygenase (EC 1.14.13.168), while 2-hydroxyphenylacetic acid can be converted to phenylacetic acid via phenylacetate-2-hydroxylase (EC 1.14.14.54). The metabolism of these plant stress hormones, coupled with the prevalence of *Tepidiformaceae* in rhizosphere samples in the EMP database (Fig. S2, Table S3–4), provides for a possibility that *Tepidiformaceae* might promote plant growth or stress tolerance. However, neither cis-jasmone nor syringic acid stimulated growth of either strain of *Tepidiforma* (Table S11), and quantitative PCR experiments at Great Boiling Spring demonstrated that *Tepidiforma* inhabits exclusively high-temperature sediments and is not a significant inhabitant of riparian plant root tissue, rhizoplane, or rhizosphere (Table S20). This does not preclude the possibility that yet-uncultivated members of the *Tepidiformaceae* could repurpose these traits for plant-growth promotion.

Several lines of evidence supported the utilization of additional substrates for growth of the new *Tepidiforma* strains. For both strains, exclusion of yeast extract or casamino acids from the medium resulted in less growth (Table S10), and incorporation of carbon from ^13^C-labeled algal amino acids was observed (Fig. 6c). Several amino acids and derivatives were also identified as substrates for growth during exometabolomics experiments (Table S12–13). This is consistent with the use of peptides and other amino acid-containing substrates like yeast extract and peptone by *T. bonchosmolovskayae* [27] and other *Chloroflexota* [34, 35, 62, 68].

Overall, the large number of compounds used by the *Tepidiforma* strains, but lack of growth on defined sole carbon sources under the conditions used here is consistent with the complex nutritional profiles of other *Chloroflexota* [34, 44, 62]. This general pattern suggests broad heterotrophic activity and metabolic interdependency with cohabiting microorganisms in nature.

## Conclusions

This study combines the isolation and characterization of two novel thermophilic *Dehalococcoidia* with a comprehensive evolutionary and comparative genomic investigation of the class. Flagellar motility and a peptidoglycan-containing cell envelope were two notable traits observed in the new strains, confirming the genus *Tepidiforma* as the sole cultivated taxon with flagellar motility in the *Chloroflexota* and peptidoglycan within the *Dehalococcoidia*. Despite the previous view that these traits are absent in the *Dehalococcoidia*/*Chloroflexota*, we show that they are, in fact, ancestral within the class, and were lost preceding the successful adaptive radiations of the *Dehalococcoidales* and the Marine Radiation that are the most widespread and abundant *Dehalococcoidia* on modern Earth. As both flagellar motility and peptidoglycan biosynthesis are energy-intensive, their loss during genome reductions, as evidenced by overall smaller genomes within these orders (Table S2), presumably lowered genetic load and increased fitness of the progenitor of this lineage in niches where flagellar motility and peptidoglycan were not selected for, namely the marine pelagos. Motility is rare among pelagic oligotrophs with small cell size due to nutrient limitations and the high effect of Brownian motion on small cells, resulting in inefficient swimming at a high energy cost [69–71]. Many modern pelagic *Dehalococcoidia* fit this paradigm because MAGs have often been recovered from small cell size fractions (<0.45 µm).

The adaptive radiation of the *Dehalococcoidia* into the oceans would have substantially altered the microbial communities inhabiting the deep sea. Rough estimates for the divergence of well-sampled classes of *Chloroflexota* range between 1000 and 1500 Mya [72], and preliminary dating analyses placed the divergence between cultivated members of the *Dehalococcoidaceae* (i.e., *Dehalogenimonas* and *Dehalococcoides*) at ~500 Mya [32, 72]. This would place the marine radiation between 500 and 1000 Mya, which is similar to marine transitions documented in other microbial lineages, particularly marine ammonia-oxidizing archaea [73, 74], and may have coincided with the Neoproterozoic Oxygenation of the oceans and associated changes in redox-active metal solubility [15, 73, 74]. However, a recent molecular dating analysis focused on diversification of prokaryotes in the ocean, placed the date of divergence of the SAR202 lineage (composed of several orders within the class *Dehalococcoidia*) prior to the Great Oxidation Event, likely during an oxygen oasis [75]. Future evolutionary studies of key marine microbiota, like the recent multi-gene dating analysis [75], should strive to refine these dates coupled with expanded taxon representation to better understand the relationship between multiple adaptive radiations of marine prokaryotes and Earth-system changes that may have enabled them.

## Protologues

### Description of *Tepidiforma flava* sp. nov

#### *Tepidiforma flava* (fla’va. L. fem. adj. *flava* yellow, the color of colonies)

This species has the following characteristics in addition to those described for the genus. Colonies grow well on R2A medium and weak on *Thermus* 162 medium and T5 agar. Colonies are small, circular, convex, and pale yellow in color on all test agar media after 3 days. The temperature range for growth is 45–65 °C, with an optimum at 55–60 °C. The pH range for growth is 6–8, with an optimum at pH 7. Growth is observed at and below 1.0% (w/v) NaCl concentration. Cells are motile, and sheared flagella were observed by cryo-electron microscopy and genomes encode genes required for flagellar biosynthesis. Cell envelopes contain a cytoplasmic membrane, peptidoglycan, and an S-layer, and cell growth is inhibited by ampicillin at concentrations ≥4 µg/ml and carbenicillin at ≥8 µg/ml. Positive for oxidase and catalase. Milk coagulation and peptonization, starch hydrolysis, cellulose hydrolysis, and H_2_S production are negative. Tweens 40 and 60 are hydrolyzed, but Tweens 20 and 80 are not. None of the following compounds can either be used as sole carbon sources or stimulate growth: azelaic acid, cis-jasmone, citrate, dextrin, D-fructose, D-galactose, D-ribose, D-xylose, fumarate, malate, maltose, mannitol, melibiose, oxalate, p-coumaric acid, propionate, pyruvate, raffinose, succinate, sucrose, and syringic acid. Acetate and vanillin cannot be used as sole carbon sources but stimulate growth, while significantly lower growth is observed with the exclusion of yeast extract and casamino acids from the growth medium. Whole-cell hydrolysates contain glucose, mannose, ribulose, galactose, and arabinose. Strictly aerobic; unable to ferment or respire anaerobically in all experiments conducted. The major fatty acid profile (>5% of total fatty acids) contains C_20:0_, C_18:0_, C_16:0_, and 10-methyl C_16:0_.

The type strain is YIM 72310^T^ ( = CGMCC 1.13591^T^ = KCTC 52670^T^), isolated from Hamazui (Frog Mouth) Hot Spring at Rehai National Park, Tengchong, Yunnan Province, south-west China. The genomic DNA G + C content of the type strain is 71.1%. The GenBank assembly accession number for the complete genome assembly is GCA_027594505.1.

### Description of *Tepidiforma thermophila* sp. nov

#### *Tepidiforma thermophila* (ther.mo’phi.la. Gr. n. *therme* heat; Gr. fem. adj. *phila* loving; N.L. fem. adj. *thermophila* heat-loving)

This species has the following characteristics in addition to those described for the genus. Colonies grow well on R2A medium and weak on *Thermus* 162 medium and T5 agar. Colonies are small, circular, convex, and pale yellow in color on all test agar media after growth for 3 days. The temperature range for growth is 45–65 °C, with an optimum at 55–60 °C. The pH range for growth is 6–8, with an optimum at pH 7. Growth is observed at and below 1.0% (w/v) NaCl concentration. Cells are motile, and genomes encode genes required for flagella biosynthesis. Cell envelopes consist of a cytoplasmic membrane, peptidoglycan, and an S-layer. Positive for oxidase and catalase. Milk coagulation and peptonization, starch hydrolysis, cellulose hydrolysis, and H_2_S production are negative. Tweens 40 and 60 are hydrolyzed, but Tweens 20 and 80 are not. None of the following compounds can either be used as sole carbon sources or stimulate growth: azelaic acid, cis-jasmone, citrate, dextrin, D-fructose, D-galactose, D-ribose, D-xylose, fumarate, malate, maltose, mannitol, melibiose, oxalate, p-coumaric acid, propionate, pyruvate, raffinose, succinate, sucrose, syringic acid, and vanillin. Acetate cannot be used as a sole carbon source but does stimulate growth. Exclusion of yeast extract from the growth medium significantly reduces growth. Whole-cell hydrolysates contain glucose, mannose, ribulose, galactose, and arabinose. Strictly aerobic; unable to ferment or respire anaerobically in experiments that were conducted. The major fatty acid profile (>5% of total fatty acids) contains C_20:0_, anteiso-C_14:0_, C_18:0_, and C_16:0_.

The type strain is G233^T^ ( = CGMCC 1.13589^T^ = KCTC 52669^T^), isolated from Great Boiling Spring in Nevada, USA. The genomic DNA G + C content of the type strain is 69.4%. The GenBank assembly accession number for the complete genome assembly is GCA_002563855.1.

## Methods

### Sample collection and strains isolation

The sample from which strain YIM 72310^T^ was isolated was collected from Hamazui (Frog Mouth) Hot Spring (HMZ; pH 7.2, temperature 68 °C), located in the Rehai Geothermal Field in Tengchong County, Yunnan Province, China, at 24°57′12.6′′N, 98°26′17.5″E. The sample from which strain G233^T^ was isolated was collected from Great Boiling Spring (GBS), located in northwestern Nevada, U.S.A., at 40°39′41′′N, 119°21′58′′W, corresponding to site E (pH 7.24, temperature 62 °C) described previously [76]. Detailed site description, sediment mineralogy, water chemistry, and microbial community composition at the sampling locations have been determined on several sampling trips and have been reported elsewhere [76, 77]. Sediment and microbial mat samples were collected with sterile spatulas and spoons, and samples were homogenized in pre-sterilized aluminum pans. Homogenized samples were dispensed into 15 ml polypropylene tubes and transported to the lab in the dark without temperature control. Once in the lab, sediment slurries were serially diluted, plated directly onto Reasoner’s 2 A (R2A) agar (containing 0.5 g proteose peptone (Difco #3), 0.5 g casamino acids, 0.5 g yeast extract, 0.5 g dextrose, 0.5 g potato starch, 0.3 g K_2_HPO_4_, 0.3 g sodium pyruvate, 0.05 g MgSO_4_.7H_2_0, and 20.0 g agar per liter deionized water), and incubated in the dark at 60 °C. Isolated colonies were re-streaked several times to obtain axenic cultures. The purified strains were routinely cultured on either R2A or a double concentration R2A supplemented with Wolfe’s Vitamin solution according to Dodsworth et al. [35], hereafter referred to as 2R2AW, at 55–60 °C and maintained as a glycerol suspension (20% v/v) at −80 °C (see Supplementary Note 1 for supplemental methods for physiological testing and DNA extraction procedures).

### Genome sequencing, assembly, and annotation

Short-read sequence data for strain YIM 72310^T^ was generated at Novogene (Novogene, Beijing, China, http://www.novogene.cn/) using a HiSeq 2000 sequencer (Illumina, San Diego, CA, USA), and resulting paired-end reads were assembled using SOAP*denovo* [78]. Long-read sequence data for strain YIM 72310^T^ was generated with Oxford Nanopore Technology (Oxford Nanopore Technologies, Oxford, UK) and assembled with Flye v. 2.8.2 [79]. The assembly generated with short-read sequence data was aligned to the long-read assembly for error correction using the “map to reference” function with default settings in Geneious v. 7.0.6 (https://www.geneious.com). The genome sequence of strain G233^T^ was generated at the Department of Energy (DOE) Joint Genome Institute (JGI) using Pacific Biosciences (PacBio) sequencing technology [80]. All raw reads for strain G233^T^ were assembled using HGAP version 2.3.0 [81]. The DNA G + C content for both strains was calculated from the genome sequences. Protein-coding sequences of both strains were annotated using multiple annotation pipelines (see Supplementary Note 1 for full methods).

### Phylogenomics and genome comparisons

To obtain a robust phylogenomic framework for the phylum *Chloroflexota*, all publicly available genomes for the phylum incorporated in the GTDB [11] release 202, were identified. Of these, the highest quality genome for species-level groups with high-quality genomes available were selected as species representatives [>90% completeness and <5% contamination estimated with CheckM [82] in GTDB], and were used for phylogenomic inference (See Table S1 for detailed genome information). This was done by identifying and aligning the bac120 phylogenetic markers through the GTDB Toolkit v. 1.4.1 (GTDB-Tk [83]). Individual alignments of markers were subjected to model testing, concatenation, and partitioning, and a maximum-likelihood phylogeny was constructed as described in the Supplementary Note 2. This process was also repeated for the class *Dehalococcoidia*, after removal of markers from the dataset that were systematically absent from the genomes within the class. Pairwise Average Nucleotide Identity (ANI) and Average Amino Acid Identity (AAI) calculations, and other comparative genomics were performed as described in the Supplementary Note 2.

### Geographic and environmental distribution

The geographic distribution of *Tepidiformaceae* and *Tepidiforma* was defined by applying the GTDB [11] release 202 taxonomy to classify 16S rRNA gene amplicon sequence variants (ASVs) from the Earth Microbiome Project (EMP) [31] release 1. For EMP data, sample processing, sequencing, and core amplicon data analysis were performed by the Earth Microbiome Project (www.earthmicrobiome.org), and all amplicon sequence data and metadata have been made public through the EMP data portal (qiita.microbio.me/emp). *Chloroflexota* genome assembly accession numbers were obtained from the GTDB and 16S rRNA gene sequences were extracted from assemblies. Sequences longer than 500 nt were retained. GTDB *Chloroflexota* 16S rRNA gene sequences were searched against SILVA [84]. *Chloroflexota* 16S rRNA gene sequences in the 138 SSU NR 99 database, and a *Chloroflexota-*specific, genome-calibrated QIIME2 [85] classifier was constructed as described in Supplementary Note 3. EMP biom files were used to obtain ASVs and resulting ASVs were classified with the *Chloroflexota* classifier (see Supplementary Note 3 for specific method). The proportion of *Tepidiformaceae* and *Tepidiforma* present in each EMP sample was displayed using the R packages ggplot v. 2 3.3.3 and cowplot v. 1.1.1.

### Light and electron microscopy

Cell morphology was investigated using cells grown on R2A at 60 °C that were harvested prior to reaching maximum turbidity. Phase-contrast micrographs of the two strains were recorded with a Zeiss Axioplan 2 imaging microscope. To obtain high-resolution images of strains YIM 72310^T^ and G233^T^, cells were imaged with cryo-electron microscopy (2D, cryo-EM) and cryo-electron tomography (3D, cryo-ET). Briefly, 1 ml of cell culture was centrifuged at 1000 × *g* for 2 min to pellet insoluble components in the media. The remaining solution was then run through a 70 μm nylon filter (Fisher Scientific) and then centrifuged at 3000 × *g* for 5 min. The resulting cell pellet was resuspended in 20 μl of cell media. Cryo-EM grids were prepared using an automated Leica EM GP plunge freezer with the sample chamber set at 21 °C and 95% humidity. 3 μl of the cell solution was pipetted onto freshly glow-discharged copper R2/2 200 grids (Quantifoil), pre-blotted for 60 s, blotted for 2 s, plunged into liquid ethane, and stored in liquid nitrogen. The grids were imaged on a 120 kV Talos L120C transmission-electron microscope at the Netherlands Center for Electron Nanoscopy (NeCEN). For cryo-ET, bacterial cultures were mixed with 20 nm colloidal gold and plunge frozen with a Vitrobot Mark IV (ThermoFisher Scientific) onto R2/2 EM grids. Tilt series were collected using SerialEM [86] on a Titan Krios 300 kV transmission electron microscope (ThermoFisher Scientific) equipped with a Falcon 34 direct electron detector. Data collection conditions were −6 µm defocus, 120 e−/Å total dose, +/− 60° oscillations, with 1° tilt increments. 3D reconstructions were calculated using IMOD with the back-weighted projection method.

To purify the sacculi, a 500 ml culture of *Tepidiforma flava* YIM 72310^T^ grown for 8 days was cooled on ice and pelleted at 5000 × *g* for 15 min at 4 °C. Cells were resuspended in 12 ml of ice-cold 100 mM NaCl and added dropwise to 10 ml 4% SDS. An additional 10 ml of 8% SDS was added to bring the final concentration to 4%. The mixture was stirred in a water bath at 80 °C for 60 min. The suspension was split evenly into two centrifuge tubes and spun at 43,000 × *g* for 30 min at 20 °C then resuspended in 40 ml ultrapure water. This wash was repeated another 5 times, then combined and resuspended in 9 ml 10 mM sodium phosphate buffer (pH 7.8). Subsequently, MgSO_4_ (10 mM) and DNase I (10 µg/ml) were added and tubes were incubated at 30 °C for 30 min without shaking, followed by addition of RNase A (20 µg/ml), and incubation for an additional 30 min with shaking at 225 rpm. Trypsin (1 mg/ml) was added, and the suspension was further incubated with shaking at 30 °C overnight. The digestion was washed with ultrapure water and pelleted at 43,000 × *g* at room temperature for 30 min. The pellet was resuspended in 10 ml 4% SDS and stirred at 95 °C for 30 min. The mixture was topped off with ultrapure water and centrifuged at 43,000 × *g* at room temperature for 30 min, then resuspended in ultrapure water and EDTA (8 mM) was added. The mixture was washed an additional four times and resuspended in 1 ml ultrapure water. The sacculi prep was stored at −80 °C prior to imaging. 4 µl of the sacculi preparation was loaded and plotted off 3 × onto Formvar caron 200 mesh copper grids, incubated for 1 min and washed 3 times with dH_2_O. Samples were stained with 0.5% uranyl acetate for 30 s and washed 3 times with dH_2_O. Negative stain TEM images were collected at ×6700 magnification on a 120 kV Talos L 120 C microscope equipped with a Ceta camera.

### Exometabolomics

Quantitative exometabolomics was performed using liquid chromatography coupled with tandem mass spectrometry (LC-MS/MS) as described previously [62]. Strains YIM 72310^T^ and G233^T^ were cultivated in 20 ml volumes of R2A in 165 ml serum bottles with an air headspace until the late-exponential phase of growth (4 days). Sterile controls of the growth medium were stored at 4 °C in the dark or were incubated along with the culture tubes to account for thermal degradation of medium components. To extract metabolites, 10 ml of the media samples were frozen and lyophilized (FreeZone 2.5 Plus, Labconco), followed by addition of 200 µl MeOH-containing 10 µM internal standard (5–50 µM of ^13^C,^15^N cell-free amino acid mixture, Sigma #767964) to each sample, brief vortexing, and water bath sonication for 10 min. After centrifuging at 3000 rcf for 5 min, samples were sonicated for an additional 5 min followed by centrifugation for a further 5 min. The supernatant was removed and centrifuge-filtered through a 0.22 µm PVDF membrane (Millipore, Ultrafree-CL GV, #UFC40GV0S) for 2.5 min at 2500 rcf, transferred to amber glass LC-MS vials, and diluted 1:9 with MeOH in preparation for LC-MS analysis.

Filtered extracts were analyzed by LC-MS/MS on an Agilent 1290 LC stack, with MS and MS/MS data collected using a Q Exactive Orbitrap MS (Thermo Scientific, San Jose, CA) with ESI source. Full MS spectra was collected from m/z 70-1050 at 70 000 FWHM resolution, with MS/MS fragmentation data acquired using 10, 20, and 30 V collision energies at 17,500 FWHM resolution. MS instrument parameters included sheath gas flow rate of 50 (au), auxiliary gas flow rate of 20 (au), sweep gas flow rate of 2 (au), 3 kV spray voltage and 400 °C capillary temperature. Normal phase chromatography was performed using a HILIC column (Millipore SeQuant ZIC-HILIC, 150 × 2.1 mm, 5 µm, # 50454) at 40 °C and using a 2 µl injection volume for each sample. The column was equilibrated with 100% buffer B (95:5 ACN:H2O w/ 5 mM ammonium acetate) for 1.5 min at 0.45 ml/min, diluting buffer B down to 65% with buffer A (100% H2O w/ 5 mM ammonium acetate) for 13.5 min, down to 0% B over 3 min while increasing flow to 0.6 ml/min and followed by isocratic elution in 100% buffer A for 5 min. Samples consisted of eight biological replicates each and three extraction controls, with sample injection order randomized and an injection blank of 100% MeOH run between each sample.

Metabolites were identified based on exact mass and retention time (RT) coupled with comparing MS/MS fragmentation spectra to that of purchased standards run using the same LC-MS methods and instrumentation (details of LC-MS/MS gradient, conditions and metabolite identifications are provided in Tables S14–S17). LC-MS data were analyzed using a custom Python code [87]. A set of criteria were used to evaluate each of the detected peaks and assign a level of confidence, indicated by a score from 0 to 3, in the compound identification. Compounds given a positive identification had matching retention time and m/z to a compound standard run using the same methods and in many cases had matching MS/MS fragmentation spectrum to either an outside database (METLIN) or collected on a Q Exactive Orbitrap MS. Metabolites given a positive identification had detected m/z ≤ 5 ppm or 0.001 Da from theoretical as well as RT ≤ 0.5 min compared to a standard run using the same LC-MS method, with the highest level of positive identification (score of 3) also having matching MS/MS fragmentation spectra. An identification was invalidated when collected MS/MS fragmentation spectra did not match the standard. Metabolites with at least one mean peak height intensity (au) > 10^5^ were analyzed statistically using a Shapiro–Wilk test (*p* > 0.05) of normality followed by a Tukey’s HSD test (*p* < 0.05), both performed using R version 3.4.3.

### ^13^C stable isotope experiments

10 ml of 2R2AW broth in 25 ml Balch tubes with an air headspace and closed with gas-impermeable aluminum seals with silicone septa were supplemented in triplicate with ^13^C-labeled (specific carbon atom labeled, or all carbon atoms labeled (U)) algal amino acids (U), xylose (U), galactose (U), ribose (U), pyruvate (1-^13^C and 2,3-^13^C), acetate (1-^13^C), lignin (U), hemicellulose (U), cellulose (U), vanillate (U), catechol (U), and ^15^N-labeled ammonium sulfate (U) at 0.1% w/v, and catechol (0.025% w/v) (99 atom fraction %; Cambridge Isotope Laboratories, Andover, MA, United States), and lignin (0.05% w/v) (97 atom fraction %; IsoLife bv, Wageningen, Netherlands). Media were inoculated with exponential-phase YIM 72310^T^ cultures at 2% v/v and incubated at 58.5 °C for 8 days. To measure ^13^CO_2_ production from ^13^C-labeled substrates, headspace samples were analyzed with cavity ring-down spectroscopy using a Picarro G2201 -i Isotopic Analyzer (Picarro Inc., Santa Clara, CA, USA) (see Supplementary Note 7 for full methods description). To measure assimilation of ^13^C-labeled substrates into microbial biomass, ^13^C isotope incorporation was measured in individual cells using the CAMECA NanoSIMS 50 at Lawrence Livermore National Laboratory, and the data were used to estimate substrate incorporation (see Supplementary Note 7 for full methods description). Killed controls of ^13^C-labeled lignin and -catechol were also included by growing YIM 72310^T^ in 2R2AW containing no labeled substrate for 8 days, followed by paraformaldehyde fixation. The killed cells were incubated an additional day with labeled lignin and catechol, then centrifuged and resuspended as before, and analyzed as described in Supplementary Note 7.

## Supplementary information

Supplementary Notes

Supplemental Figures

Supplemental Tables

Supplemental data file S1

## Data Availability

Genome sequence data for the two novel isolates have been submitted to the National Center for Biotechnology Information (NCBI) and are available under the GenBank assembly accession numbers GCA_027594505.1 (*Tepidiforma flava* YIM 72310^T^) and GCA_002563855.1 (*Tepidiforma thermophila* G233^T^). For exometabolomics, all raw data is available under the MassIVE dataset MSV000090480 accessible through GNPS at https://massive.ucsd.edu/ProteoSAFe/dataset.jsp?task=895e11f85bac44f2828880c77ff4ef8f, and metabolite identification data are available under the FigShare collection “Exometabolomics analysis for MassIVE dataset MSV000090480” at 10.6084/m9.figshare.c.6236283.v1. All other data are included as supplemental material to this paper.
