# Supplementary material for: Thermophilic Dehalococcoidia with unusual traits shed light on an unexpected past
Source: ISME J. 2023 Apr 11;17(7):952–66. doi: 10.1038/s41396-023-01405-0 (PMC10284905; doi:10.1038/s41396-023-01405-0)
Supplement: Supplementary file 1 — Supplementary Notes [file 41396_2023_1405_MOESM1_ESM.docx]

**Thermophilic *Dehalococcoidia* with unusual traits shed light on an unexpected past**

Marike Palmer, Jonathan K. Covington, En-Min Zhou, Scott C. Thomas, Neeli Habib, Cale O. Seymour, Dengxun Lai, Juliet Johnston, Ameena Hashimi, Jian-Yu Jiao, Alise R. Muok, Lan Liu, Wen-Dong Xian, Xiao-Yang Zhi, Meng-Meng Li, Leslie P. Silva, Benjamin P. Bowen, Katherine Louie, Ariane Briegel, Jennifer Pett-Ridge, Peter K. Weber, Elitza I. Tocheva, Tanja Woyke, Trent R. Northen, Xavier Mayali, Wen-Jun Li, Brian P. Hedlund

**Supplementary Notes** Page

Supplementary Note 1: DNA extraction, sequencing, assembly, and annotation 1

Supplementary Note 2: Phylo- and comparative genomics of the *Dehalococcoidia* 4

Supplementary Note 3: Geographic and environmental distribution 6

Supplementary Note 4: Additional information on cell morphology 7

Supplementary Note 5: Evolutionary history of unusual traits 8

Supplementary Note 6: Cultivation experiments and genomics 16

Supplementary Note 7: ^13^C stable isotope experiments 19

**Supplementary Notes**

***Supplementary Note 1: DNA extraction, sequencing, assembly, and annotation***

High molecular weight genomic DNA was extracted from cell pellets of 150 ml cultures grown in R2AW, using a modified version of the JGI bacterial CTAB extraction protocol (https://jgi.doe.gov/user-programs/pmo-overview/protocols-sample-preparation-information/jgi-bacterial-dna-isolation-ctab-protocol-2012/). Modifications to the protocol included three freeze-thaw cycles following the lysozyme treatment, in a dry ice/ethanol bath and a water bath at 65 °C for three minutes per cycle, and the use of RNase A (Promega, Madison, WI, USA, catalogue number: A7973) for denaturation of ribonucleic acids. For the generation of short-read Illumina sequence data, the genome of strain YIM 72310^T^ was sequenced with a HiSeq 2000 platform. Long-read sequence data for strain YIM 72310^T^ was generated with Oxford Nanopore Technology (Oxford Nanopore Technologies, Oxford, UK) on a MinION Mk1B device, using the EXP-NBD104 Native Barcoding Expansion Kit and the SQK-LSK109 Ligation Sequencing Kit, following the manufacturer’s instructions. A hybrid genome assembly using Illumina short-read data for error correction of the MinION long-read data was generated for YIM 72310^T^. A total of 38,638 long reads passed sequencing quality control, totaling 375.4 Mbp of sequence data. For the G233^T^ genome, a PacBio SMRTbell^TM^ library was constructed and sequenced on the PacBio RS platform. From this sequencing run, 188,108 filtered reads totaling 422.6 Mbp were used to assemble the genome.

The genome of strain G233^T^ comprised 2,743,337 bp in one scaffold with a GC content of 69.4%. The genome of strain YIM 72310^T^ comprised 2,767,986 bp in one scaffold with an average GC content of 71.1%. Both genomes of the novel species were similar in size to that of *Tepidiforma bonchosmolovskayae* (2.7 Mb) and were markedly larger than genomes from other cultivated members of the *Dehalococcoidia*, e.g., *Dehalococcoides mccartyi* 195^T^ (1.47 Mb) and *Dehalogenimonas lykanthroporepellens* BL-DC-9^T^ (1.69 Mb). The G+C content of both isolates were also in a similar range to the 69.9% of *T. bonchosmolovskayae* (Kochetkova et al., 2020), while the G+C content of *Dehalococcoides mccartyi* and *Dehalogenimonas lykanthroporepellens* were lower, at 48.9% and 55%, respectively.

Genome annotation for both genomes was performed through homology searches against the Kyoto Encyclopedia of Genes and Genomes (KEGG) database (<https://www.genome.jp/kegg/kegg1.html>), using the KofamKOALA search algorithm (Aramaki et al., 2020), in order to better understand the metabolism of the strains and make predictions about their physiologies. The genomic sequence data of *T. bonchosmolovskayae* 3753O^T^ and additional genomes belonging to the order *Tepidiformales* (o__UBA2991 in **Table S2**) were obtained from the National Center for Biotechnology Information (NCBI; <https://www.ncbi.nlm.nih.gov/>). All genomes were reannotated using default settings with the RAST (Rapid Annotation using Subsystems Technology) toolkit v. 0.1.1 (Overbeek et al., 2014; Brettin et al., 2015), as implemented in KBase (Arkin et al., 2018), in order to limit inconsistencies caused by differences in annotation pipelines. All genomes analyzed were also subjected to broad functional annotation using eggNOG-mapper v. 2 (Cantalapiedra et al., 2021).

A total of 2,764 genes were predicted for G233^T^ with RAST toolkit v.0.1.1 in KBase, comprising 2,715 protein-coding genes and 49 RNA genes, while a total of 2,913 genes were predicted for YIM 72310^T^, comprising 2,863 protein-coding and 50 RNA genes (**Table S12, S13**). This is comparable to the 2,717 protein-coding genes predicted for *T. bonchosmolovskayae* (Kochetkova et al., 2020). Both strains had high proportions of genes assigned to COG functional categories involved in the transport and metabolism of lipids (6.5% of genes for strains G233^T^ and YIM 72310^T^, respectively), slightly lower than the 8.9% of genes encoded by *T. bonchosmolovskayae* (Kochetkova et al., 2020). Genes assigned to COG categories associated with coenzymes (4.7% of genes of both novel strains, compared to 7.5% in *T. bonchosmolovskayae*), and amino acids (6.8% of genes encoded by both novel strains compared to 8.3% in *T. bonchosmolovskayae*) were also proportionally high, while those associated with the metabolism of nucleotides (2.7% of genes compared to 2.3% in *T. bonchosmolovskayae*) were markedly lower. Overall, these results were comparable to those reported for *T. bonchosmolovskayae* (Kochetkova et al., 2020).

Both G233^T^ and YIM 72310^T^ had similar metabolisms as predicted by KEGG, including complete tricarboxylic acid cycles as well as intact pathways for glycolysis, the non-oxidative pentose-phosphate pathway, and gluconeogenesis (**Table S12** and **S13**). Most enzymes of the glyoxylate cycle were present, but neither of the key enzymes isocitrate lyase nor malate synthase were annotated. Both strains possessed conserved pathways for the synthesis of pantothenate, Coenzyme A, purines and pyrimidines, and nearly complete pathways for synthesis of riboflavin. Modules for synthesis of amino acids were predicted, although the annotated histidine biosynthetic pathway was incomplete. A full carotenoid biosynthetic pathway was present, with predicted capacity for synthesis of phytoene, zeta-carotene, and lycopene, consistent with the suggestion that yellow pigments in *T. bonchosmolovskayae* are carotenoids (Kochetkova et al., 2020). A beta-oxidation pathway, including acetyl esterase/lipase, was identified in both genomes. The genomes both encoded complete flagellar gene clusters, chemotaxis systems, and peptidoglycan biosynthetic pathways (see main manuscript for details).

The genomes of G233^T^ and YIM 72310^T^ encoded 21 putative ABC transporters, as predicted by KofamKOALA, with complete pathways for transport of phosphate, branched-chain amino acids, and oligopeptides. KEGG analysis also revealed that G233^T^ possessed an additional enzyme for the conversion of (S)-3-hydroxy-3-methylglutaryl-CoA into 3-methyl-glutaconyl-CoA during valine and leucine degradation. Additionally, the genomes of both novel strains contained two operons predicted to encode distinct 11-subunit NADH-ubiquinone oxidoreductase isozymes (Nuo, complex I), while they lacked genes for a cytoplasmic electron input “N-module”, i.e., NuoEFG subunits (**Table S12** and **S13**). Although the physiological contribution of a second complex I isozyme is not clear, its association with organisms that are energetically and physiologically versatile is consistent with the hypothesis that individual isozymes may perform different functions or could be important for different modes of growth (Spero et al., 2016). Like *T. bonchosmolovskayae,* these strains have biosynthetic pathways for the low-affinity, high velocity cytochrome c oxidase (respiratory complex IV). Subunits I and II of the high-affinity cytochrome bd ubiquinol oxidase were also present. The noncatalytic accessory proteins, CydX or AppX, were not annotated.

***Supplementary Note 2: Phylo- and comparative genomics of the* Dehalococcoidia**

Individual alignments of the marker sequences used in the Bac120 dataset were subjected to model testing and concatenation using FASconCAT-G v. 2 (Kück and Longo, 2014) and ProtTest v. 3.4 (Darriba et al., 2011), using the Akaike Information Criterion. A maximum-likelihood phylogeny for the *Chloroflexota* was inferred with IQ-Tree v. 1.3.11.1 (Nguyen et al., 2015) from the concatenated, partitioned matrix, allowing partition-specific evolutionary rates (Chernomor et al., 2016). Branch support was inferred with the Ultrafast Bootstrap algorithm (Hoang et al., 2018), and the Shimodaira-Hasegawa-like approximate likelihood ratio test (SH-aLRT; (Guindon et al., 2010)), from 1,000 replicates each. The generated trees were visualized by iTOL v.3 (Letunic and Bork, 2021) or MEGA-X (Kumar et al., 2018), and edited in Inkscape v. 0.92.

Overall, highly-supported robust phylogenomic trees were reconstructed for the *Chloroflexota* and the class *Dehalococcoidia*. The full *Chloroflexota* phylogenomic tree, including all 694 high-quality genomes included in the GTDB release 202, allowed recovery of most GTDB designated classes as monophyletic, specifically the *Anaerolineae*, *Chloroflexia*, *Dehalococcoidia*, *Ktedonobacteria*, Ellin6529, FW602-bin22, UBA4733, and UBA6077, with UBA11872 nested in the class UBA2235, with SH-aLRT branch support > 0.95 (black dots in **Fig. S1**). In contrast to the current taxonomic structure for the phylum presented in literature, as reflected by the names with standing in prokaryotic nomenclature, the validly published classes *Ardenticatenia*, *Caldilineae*, and *Thermoflexia* were all nested as orders in the class *Anaerolineae*, while the validly published class *Thermomicrobia* was nested within the class *Chloroflexia* as an order, and the validly published class *Tepidiformia* being nested in the class *Dehalococcoidia* as an order.

Furthermore, Average Amino acid Identity (AAI) and Average Nucleotide Identity (ANI) comparisons were performed to obtain an indication of relatedness among the members of *Tepidiformales* based on Overall Genome Relatedness Indices (OGRI). Both indices were calculated on the Enveomics platform with Genome Matrix (<http://enve-omics.ce.gatech.edu/g-matrix/index>; (Rodriguez-R and Konstantinidis, 2016)). For further comparative genomics, all genomes for the *Tepidiformales* were used to build a pangenome for the order *Tepidiformales* using OrthoMCL v. 2.0 (Li et al., 2003) with *T. bonchosmolovskayae* as the base genome.

Among the three cultivated representatives of the genus *Tepidiforma*, AAI and ANI values were consistent with distinct species (**Fig. 1A**), with *T. bonchosmolovskayae* being the closest relative to *T. thermophila*, followed by *T. flava* splitting off at the most basal node within the genus. Similarly, two other lineages consistent with genus-level OGRI diversity, designated HRBIN29 and UBA2991 by the GTDB, and the singleton belonging to the designated genus SLAK0 each shared AAI values between 45% and 65% among the lineages and indicates a likely familial relationship among them. The current known diversity for the order *Tepidiformales* thus currently corresponds to a single family, consisting of at least four genera (**Fig. 1A**).

To investigate the potential functions of shared genes among members of *Tepidiformales* and *Tepidiforma*, gene annotations obtained with different annotation pipelines were compared. The core genome based on the three *Tepidiforma* isolates consisted of 2,371 genes, of which 306 genes were *Tepidiforma*-specific (**Table S5**). Of these 306 genes, approximately 3% were associated with putative ABC transporters, although, among the different annotation pipelines, the specific transporters encoded by these genes were not always consistent (**Table S5**), likely due to low similarity between these genes and those of well-characterized organisms. Furthermore, 99 genes annotated as encoding hypothetical proteins, and a further 123 genes with no functional annotations were shared among all three members of *Tepidiforma*.

All *Tepidiformales* genomes encode multiple alternative *ftsI* homologs, which could mediate resistance to beta-lactam antibiotics, although strain YIM 72310^T^ was still susceptible to concentrations above 4 µg/ml of ampicillin, and 8 µg/ml of carbenicillin (**Fig. S3).** Many genes associated with oxidative phosphorylation were also conserved (**Table S5**), including cytochrome c oxidase and cytochrome bd ubiquinol oxidase, providing evidence for conservation of aerobic respiration in the order. Robustly identified (with at least two approaches) ABC transporter subunits shared across the group included iron, cobalt/nickel, branched-chain amino acid, molybdate, tungstate (TupA/TupB), and phosphate transporter subunits (**Table S8**). Tungstate could potentially be used as a cofactor for several annotated aldehyde ferredoxin oxidoreductases, some of which are *Tepidiforma*-specific and others of which are conserved throughout the order. However, tungstate did not affect growth at any tested concentration (**Table S18**). The order core gene set also included assimilatory arsenate reductase (ArsC) and arsenite methyltransferase, both known to be important detoxification systems in prokaryotes (Yan et al., 2019). Many geothermal systems have high arsenic concentrations, including the Rehai Geothermal Field and Great Boiling Spring (Costa et al., 2009; Guo et al., 2017). Although this may be an interesting angle for future studies into the detoxification of arsenate, no growth under anaerobic conditions occurred with either novel strain with the addition of arsenate (no statically significant difference compared to no air controls), suggesting that arsenic can likely not be used as a terminal electron acceptor for anaerobic respiration (**Table S6**).

***Supplementary Note 3: Geographic and environmental distribution***

*Chloroflexota* assemblies were retrieved from NCBI, and using BLASTN (Camacho et al., 2009) 2.9.0+, 16S rRNA gene amplicon sequence variants (ASVs) from the Earth Microbiome Project (EMP) were classified based on the GTDB taxonomy. Greater than 97% identity matches between sequences were retained. To calibrate the GTDB taxonomy to SILVA 16S rRNA gene sequences, the rank of o_UBA2991;f_UBA2991 (*Tepidiformales*;*Tepidiformaceae*) was assigned to all descendants of the MRCA of the SILVA 16S rRNA gene sequences with the highest bit-score match to each 16S sequence from o_UBA2991 genomes. These and other 16S rRNA sequences from *Chloroflexota* genomes in the GTDB were used to construct a *Chloroflexota*-specific, genome-calibrated Qiime2 (Bolyen et al., 2019) naiive-Bayesian (Bokulich et al., 2018) taxonomic classifier via Qiime2 version 2020.8. Biom-format files comprising the Earth Microbiome Project (EMP) release 1 were obtained via ftp from qiita.ucsd.edu. The EMP data are classified into three EMP ontology (empo) levels. The first empo level groups data into free-living or host-associated samples. The second empo level groups free-living samples into saline or non-saline environments, and host-associated samples into animal or plant associated. The third empo level consist of more specific environmental ontologies found in each of these larger categories, e.g., non-saline subsurface samples group into non-saline samples at empo level 2 and as free-living at empo level 1. These files were converted to plain text using biom (McDonald et al., 2012) 2.1.10 and split into DNA sequence fasta files with corresponding long-format ASV matrices in R version 3.6.3. All ASV representative sequences were truncated to 90 nt. Sequence files and ASV matrices were then concatenated vertically. Duplicated sequences, including those which were identical to another only after truncation, were collapsed into single ASVs. Dereplicated sequences were classified using a naiive-Bayesian taxonomic classifier trained on the entire SILVA 138 SSU NR 99 database. Sequences classified as Eukaryotic and those unclassified at the domain level were removed. Sequences classified as *Chloroflexi* according to the SILVA classifier were reclassified using the *Chloroflexota*-specific classifier.

***Supplementary Note 4: Additional information on cell morphology***

Cells of strains YIM 72310^T^ and G233^T^ were comparable to cells of *T. bonchosmolovskayae* (**Fig. 2**) (Kochetkova et al., 2020). Cells of both new strains were rod-shaped with distinctive conical ends with dimensions of 1.0-2.5 µm by 0.2-0.4 µm. Both divided by formation of a transverse septum, and contained intracellular polyphosphate granules, typically near the cell poles. The cell envelopes of both strains were composed of thin layers, similar to *T. bonchosmolovskayae*. In all three species, the inner layer is most likely the cytoplasmic membrane, while outer layers are most likely a thin peptidoglycan layer and a glycoprotein S-layer. The cells stained Gram-negative, like many *Chloroflexota* (Yamada et al., 2006; Cole et al., 2013; Dodsworth et al., 2014). Some other *Chloroflexota* contain thin layers of peptidoglycan but stain Gram-positive (Sorokin et al., 2014). Flagellar motility was observed occasionally in cultures and confirmed by electron microscopy (**Fig. 2**), consistent with genomic interpretations (**Table S12, S13**). Motility is mediated by a single polar flagellum in both new strains, as well as in *T. bonchosmolovskayae* (Kochetkova et al., 2020).

Consistent with the previously observed granules found in *T. bonchosmolovskayae* cells, cells of both novel strains contained granules situated at cell division planes (**Fig. 2A-C**). The presence of genes involved in polyphosphate synthesis and degradation (Wang et al., 2018), which would be consistent with these granules comprising polyphosphate, were encoded by all *Tepidiforma* genomes (**Table S12**, **S13**, and (Kochetkova et al., 2020)). Specifically, *ppk1* (polyphosphate kinase 1), *ppk2* (polyphosphate kinase 2), *gppA* (exopolyphosphatase), and *ppnK* (NAD kinase) were all annotated and present in the three species of *Tepidiforma*, while homologs of *pap* (polyphosphate:AMP phosphotransferase), *surE* (5′/3′-nucleotidase), and *ppgK* (polyphosphate glucokinase) could not be identified from the genomes of either novel strain or *T. bonchosmolovskayae* (BLASTP; E-value <1e-10).

***Supplementary Note 5: Evolutionary history of unusual traits***

To ensure consistency in annotations, all high-quality genomes belonging to the *Dehalococcoidia* were subjected to gene calling using Prodigal v. 2.6.2 (Hyatt et al., 2010) and annotation using eggNOG-mapper v. 2 (Cantalapiedra et al., 2021). From here, all KO numbers associated with peptidoglycan biosynthesis, aromatic compound degradation, and flagellar motility were identified, and used to construct a presence/absence matrix for the class. As more than 30 species-level groups consisted of multiple genomes, majority-rule consensus presence and absence profiles were generated for these species. Overall, this resulted in a presence/absence matrix for 140 species-level groups within the *Dehalococcoidia* for the respective KO terms. This allowed identification of core sets of genes that were consistently present in those genomes encoding these pathways, and the presence of these core sets of genes were plotted against a cladogram (**Fig. 3**, **Fig. 5**, and **Fig. S7**) inferred from the circular *Dehalococcoidia* phylogeny (**Fig. 1B**).

In order to infer likely evolutionary events linked to peptidoglycan biosynthetic pathways, aromatic compound degradation and flagellar motility, ancestral character state reconstructions were performed. The cladogram referred to above served as the primary evolutionary hypothesis for the class. The majority-rule consensus presence/absence profiles for all species within the *Dehalococcoidia* were then used to reconstruct potential evolutionary events (i.e., gene births, HGT, duplications, or losses) by inferring the history of gene families through rate optimization and determined by posterior probabilities, as implemented in COUNT (Csűös, 2010). The resulting gains and losses were summarized by core gene sets on the cladograms for the respective traits.

In peptidoglycan biosynthetic pathways found in the class, genes associated with DAP-type peptidoglycan synthesis pathways could largely be grouped into three core groups. The first group of genes, referred to here as Group 1 genes, encoded enzymes MurABCDEFG, MraY, MrcB, MrdA, FtsI, SpoVD, PbpA and GatD [associated with Lys-type peptidoglycan biosynthesis] (purple enzymes in pathway in **Fig. 5B**). This gene set was either absent from *Dehalococcoidia* genomes (orders 10 to 25 in **Fig. 5A**), or more than 85% of these genes were present in the genomes of any given species (orders 1 to 9 in **Fig. 5A**). This clear separation between genomes encoding these proteins and those that do not, consistently resulted in hypothesized losses for all genes in this group in the ancestral node to orders 10 to 25. Incidentally, the orders recovered as monophyletic at this node also represents the previously defined terrestrial and marine clades, including the *Dehalococcoidales*, and SAR202 lineages. The second set of peptidoglycan biosynthesis genes, referred to here as Group 2 genes, included those coding for Ddl, BacA, BcrC and UppS (pink enzymes in pathway in **Fig. 5B**). Interestingly, genes coding for these enzymes were pervasive within the class, with numerous species containing homologs to all four of these genes. The third group of genes (grey in **Fig. 5B**) were made up of MurE (associated with Lys-type peptidoglycan biosynthesis), VanY, and DacACD, and were also intermittently present in orders 1 to 9, but systemically absent from all genomes in orders 10 to 25. Although, none of the environmental genomes in the Marine Radiation are complete (not assembled to replicon-level), the consistent absence of Group 1 and 3 genes in these lineages are notable. This result does however support the likely presence of genes coding for a complete peptidoglycan biosynthetic pathway in the ancestor to the class, with a large subsequent loss occurring at the ancestor to orders 10 to 25 (including the *Dehalococcoidales* and the Marine Radiation).

For metabolic pathways associated with the degradation of aromatic compounds, a much more sporadic distribution within the class was observed (**Fig. S7**). Overall, the only KEGG pathways with several genes annotated as being present within the class were those involved in benzoyl-CoA degradation, catechol meta-cleavage, and trans-cinnamate degradation (**Fig. S7**). No clear patterns with high confidence could be discerned from the data, as the pathways for catechol meta-cleavage and trans-cinnamate degradation were never annotated as complete (**Fig. S7**), and where numerous genes associated with these pathways were annotated, no clear pattern based on evolutionary relatedness was observed. In contrast to this, three genomes within the *Dehalococcoidales* were annotated as encoding the full degradation pathway for benzoyl-CoA (**Fig. S7**), however, components of this pathway in the genomes of this class were also scattered across the phylogeny, providing limited to no support for any one evolutionary hypothesis for these genes.

To investigate the evolutionary history of genes associated with the benzoyl-CoA, catechol meta-cleavage and trans-cinnamate degradation pathways, phylogenetic analyses were conducted on genes coding for subunits of several key enzymes. Sequences analyzed included benzoyl-CoA reductase (B subunit, K04113), catechol-2,3-dioxygenase (K00446 and K07104), and trans-cinnamate dioxygenase (K05710). Peptide sequences from the *Dehalococcoidia* genomes annotated with the relevant KEGG orthology terms were compared to the non-redundant protein database of the National Center for Biotechnology Information (NCBI) using default settings with BLASTP, limiting the number of reported hits to 1,000. In the case of benzoyl-CoA reductase subunit B, functionally characterized and verified homologs belonging to the FldB/FldC dehydratase alpha/beta subunit (IPR010327) protein family, and all known homologs of the benzoyl-CoA reductase, subunit B (IPR011955) subfamily were also added to the dataset. For catechol-2,3-dioxygenase, all characterized and verified homologs belonging to the catechol-2,3-dioxygenase (IPR017624) family and the glyoxalase/bleomycin resistance/dihydroxybiphenyl dioxygenase (IPR029068) protein superfamily were also included in the dataset. For trans-cinnamate dioxygenase, all characterized and verified homologs of 3-phenylpropionate/cinnamic acid dioxygenase, ferredoxin subunit (IPR023739) family, and the Rieske [2Fe-2S] iron-sulphur domain (IPR036922) protein superfamily was included. Obtained datasets were aligned with the online MAFFT server (https://mafft.cbrc.jp/alignment/server/; (Katoh and Standley, 2013)) using the FFT-NS-1 algorithm. These aligned sequences were then trimmed with a 75% gap filter (i.e., alignment positions with more than 75% of taxa lacking sequence information was excluded) with MSA Trimmer (https://github.com/LKremer/MSA_trimmer), followed by maximum-likelihood phylogenetic inferences with IQ-Tree v. 1.3.11.1 (Nguyen et al., 2015), estimating the best-fit evolutionary model (Kalyaanamoorthy et al., 2017) for each sequence, and inferring branch support from 1,000 replicates each, with ultrafast bootstrapping (Hoang et al., 2018) and Shimodaira-Hasegawa-like approximate likelihood ratio tests (SH-aLRT; (Guindon et al., 2010)). These phylogenetic analyses were done on both the trimmed and untrimmed datasets. Trees were visualized in FigTree v. 1.4.4 and edited in Inkscape v. 0.92.4. To identify closest phylogenetic neighbors to the *Dehalococcoidia* sequences, functionally verified homologs and taxonomic assignments to medium- and high-quality genomes in the GTDB was used.

For all sequences analyzed, *Dehalococcoidia* sequences failed to group with characterized homologs of the key genes associated with annotated aromatics degradation pathways and were typically not recovered as monophyletic groups. The phylogenies reconstructed from the trimmed and untrimmed datasets were largely congruent. In the case of the benzoyl-CoA reductase sequences, BLAST hits consistently showed higher homology to other 2-hydroxyacyl-CoA dehydratase than to known benzoyl-CoA reductase subunit B sequences. Several enzyme subunits for both proteins are homologous (Buckel et al., 2014), and are classified into the InterPro family IPR010327, with the B subunit of benzoyl-CoA reductase classified into subfamily IPR011955. Due to the inconsistencies associated with the annotation of *Dehalococcoidia* sequences and BLAST hits in the non-redundant database, the dataset was supplemented with all sequences in the InterPro IPR011955 (benzoyl-CoA reductase subunit B) subfamily (241 sequences), and functionally verified and reviewed sequences in the IPR010327 protein family (11 additional sequences). This resulted in a dataset comprising 1,551 taxa, with three larger groups being recovered for this protein family (**Fig. S7**, **S8A**, and **File S1**).

The first group consisted of benzoyl-CoA reductase subunit B sequences, together with functionally characterized copies of 2-hydroxyglutaryl-CoA dehydratase subunit A, lactoyl-CoA dehydratase subunit A, phenyllactyl-CoA dehydratase subunit A and 2-hydroxyisocaproyl-CoA dehydratase subunit A. Several *Anaerolineae*, in the *Chloroflexota*, sequences were found to group with this larger group of enzymes, but no *Dehalococcoidia* sequences grouped with these sequences. The second group consisted of functionally characterized copies of phenyllactyl-CoA dehydratase subunit B, 2-hydroxyisocaproyl-CoA dehydratase subunit B, lactoyl-CoA dehydratase subunit B, 2-hydroxyglutaryl-CoA dehydratase subunit B and benzoyl-CoA reductase subunit C. Several *Dehalococcoidales* sequences were recovered as part of this larger group and grouped as sister to the *Bathyarchaeia* in the archaeal phylum *Thermoproteota*, although no discrete groupings with a particular functionally characterized homolog was observed. The third group consisted solely of unknown lineages, generally annotated as 2-hydroxyacyl-CoA dehydratases. Within this group the majority of *Dehalococcoidia* sequences grouped with either the *Desulfobacterota* (*syn.* Deltaproteobacteria) or the *Thermoproteota*, with lineages found in several *Dehalococcoidia* orders, typically representing paraphyletic groups with other phyla nested within the *Dehalococcoidia* subtrees. Thus, none of the *Dehalococcoidia* sequences annotated as encoding the benzoyl-CoA reductase subunit B grouped with sequences confidently identified as IPR011955, although limited taxon sampling in this analysis and a lack in functional data limits our ability to support the annotation of these genes as benzoyl-CoA reductase subunit B. Several unknown lineages within the IPR010327 superfamily, where *Dehalococcoidia* were pervasive (~25 % of sequences), were recovered. However, although homologs belonging to the *Anaerolineae* were identified with the BLAST searches, these sequences consistently grouped with homologs belonging to the *Firmicutes*, while the *Dehalococcoidia* sequences annotated as benzoyl-CoA reductase largely grouped with *Desulfobacterota* (*syn.* Deltaproteobacteria) and *Thermoproteota* sequences, providing some support for several horizontal transfer events to and from the *Chloroflexota*, particularly in the *Dehalococcoidia* (**File S1**). Additionally, several enzymes in the FldB/FldC dehydratase alpha/beta subunit (IPR010327) protein family are active on aromatic compounds and heterocycles, e.g., benzoyl-CoA reductase, and phenyllactyl-CoA dehydratase. Thus, although the data cannot unequivocally support these *Dehalococcoidia* homologs as involved in degradation of aromatic compounds, several of the unknown lineages may represent novel protein subfamilies associated with aromatics degradation.

For the analysis of the annotated catechol-2,3-dioxygenase (K00446 or K07104), a dataset comprising 3,324 taxa were constructed. *Dehalococcoidia* sequences annotated as encoding K00446 or K07104 typically grouped with unknown homologs from diverse phyla (**Fig. S7**, **S8B**, and **File S1**). Several lineages within the glyoxalase/bleomycin resistance/dihydroxybiphenyl dioxygenase (IPR029068) protein superfamily phylogeny were represented by sequences from the *Dehalococcoidia* orders UBA3495, SAR202, UBA2963, and SM23-28-2, with three of the lineages having *Pseudomonadota* sequences as closest relatives. Of the *Dehalococcoidia* sequences grouping with homologs of the protein superfamily with known functions, sequences from unknown *Dehalococcoidia* (too low quality to include in our taxonomic analyses) and UBA3495 grouped with glutathione transferase FosA homologs, sequences from UBA3495, UBA2963 and unknown *Dehalococcoidia* grouped with biphenyl-2,3-diol-1,2-dioxygenase, and sequences from SpSt-223 grouped with characterized catechol-2,3-dioxygenase from *Bacillus subtilis*, along with some *Anaerolineae* sequences. All other functionally verified catechol-2,3-dioxygenases/ metapyrocatechases grouped with other diverse superfamily members in an unrelated group to any *Dehalococcoidia* sequences. As all phylogenetic groups obtained in this phylogeny only contained sequences from a limited number of orders within the class *Dehalococcoidia* and were largely not congruent with the species tree (**Fig. 1**), these genes were likely all horizontally acquired, with gene transfers occurring within the *Dehalococcoidia* upon introduction to some of these orders. Further analysis coupled with functional characterization of these lineages would be required to determine the function of the novel lineages belonging to the glyoxalase/bleomycin resistance/dihydroxybiphenyl dioxygenase (IPR029068) protein superfamily.

The Rieske [2Fe-2S] iron-sulphur domain (IPR036922) protein superfamily, which includes trans-cinnamate dioxygenase, is very large and well characterized, and the dataset consisted of 5,632 sequences, with >250 functionally characterized homologs. Despite the array of characterized proteins in this superfamily, several lineages of *Dehalococcoidia* sequences grouped distantly related to any known sequences. Although several lineages contained sequences from multiple closely related orders within the class, it remains unclear whether any of these homologs were present within the ancestor to the class and vertically inherited, due to overall poor branch support and very long branches, which could result in artefactual groupings. For the lineages comprising only sequences belonging to a single order (e.g., *Tepidiformales* sequences), horizontal acquisition is the most likely scenario, although close relatives to these homologs serving as potential donors for horizontal transfer events could not be identified. Furthermore, some lineages appeared to have been acquired at a particular node within the tree (**Fig. 1**), and subsequently disseminated to other orders within the *Dehalococcoidia*. For example, one novel homolog lineage (**Fig. S8C**) included sequences from orders UBA2979 (order 3), Bin125 (order 4), DSTF01 (order 6), and *Tepidiformales* (order 8), and UBA3495 (order 18) within the marine radiation, which may be indicative of a transfer to this order. Thus, based on these analyses, horizontal gene transfer of these genes appear rampant and further analysis would be required to clarify the complex evolutionary history of aromatic degradation within the class.

As flagellar motility was observed in the novel *Dehalococcoidia*, the presence of genes coding for flagella was investigated within the class. Overall, genes were separated into those coding for structural components of the flagellar machinery (Group 1 teal-colored proteins, **Fig. 3B**), typically co-occurring when present, and genes encoding regulatory proteins (Group 2 green-colored proteins, **Fig. 3B**). Group 1 included genes coding for FliACDEFGILMNPQRS, FlgBCDEFGKL, FlhAB, and MotAB. Group 2 genes included those coding for FlgN, FliY, FlrC, and RpoDN. Other genes that were intermittently present coded for FliHJKO, FlgA and MotX. Broadly, genomes encoding the structural set of flagellar genes were either present as sets consisting of more than 90% of the Group 1 genes (at least 23 out of 25 genes), or largely absent from the genomes. However, two or more Group 1 genes (but less than 23 genes) were found scattered across the class, with several marine-associated orders having genomes with some Group 1 genes present. RpoD was also encoded by almost all genomes in the order, although several genomes across the class also encoded other regulatory proteins associated with flagellar motility. This distribution was further interrogated with ancestral character state reconstruction, which supported the likely presence of the full Group 1 set of genes in an ancestor to the class. Subsequently, these genes were likely vertically inherited and maintained in the representatives of orders 1 and 2 and the ancestor to the orders 3 to 9. However, a full loss of Group 1 genes at the node ancestral to the *Dehalococcoidales* and the marine radiation was likely. Within the early-branching lineages associated with diverse environments, a partial loss of the Group 1 genes was also predicted for the ancestor to orders 3 to 6. Similar to the losses accompanying the marine radiation, those members of the *Tepidiformales* from marine environments also had a likely partial loss in Group 1 genes.

In order to obtain evolutionary hypotheses for the origin of motility within the class, phylogenetic analyses were conducted on representative sequences encoded for by each of the flagellar gene clusters. Peptide sequences for each of the cluster representatives were compared to the non-redundant protein database of the National Center for Biotechnology Information (NCBI) using default settings with BLASTP, limiting the number of reported hits to 1,000. Obtained datasets were aligned with the online MAFFT server (<https://mafft.cbrc.jp/alignment/server/>; (Katoh and Standley, 2013)) using the MAFFT-DASH algorithm (Rozewicki et al., 2019), bringing homology in protein structures into account during alignment. These aligned sequences were then subjected to maximum-likelihood phylogenetic inferences with IQ-Tree v. 1.3.11.1 (Nguyen et al., 2015), estimating the best-fit evolutionary model (Kalyaanamoorthy et al., 2017) for each sequence, and inferring branch support from 1,000 replicates each, with ultrafast bootstrapping (Hoang et al., 2018) and Shimodaira-Hasegawa-like approximate likelihood ratio tests (SH-aLRT; (Guindon et al., 2010)) (Supplementary File S2). Trees were visualized in FigTree v. 1.4.4 and edited in Inkscape v. 0.92.4. For taxonomic assignments, medium- and high-quality genomes classified in the GTDB were used to identify phyla.

Selection criteria for representative genes included conserved function (to ensure sufficient primary sequence conservation to robustly identify homologs), large size (to ensure sufficient phylogenetic signal is captured for robust phylogenetic inference), and presence as part of the core flagellar set (**Fig. 3B**). This resulted in the selection of flagellin, *fliC*, as the Cluster 1 representative, because flagellin genes are typically well-conserved and encode proteins of greater than 500 amino acids (alignment including 581 taxa). Similarly, due to the integral role of the flagellum-specific ATP synthase and the longer length of these genes (encoding proteins of ~ 500 amino acid residues), *fliI* was selected as representative for Cluster 2 (alignment including 539 taxa). For Cluster 3, *motA* [part of the flagellar motor complex; encoding a protein of *ca.* 300 amino acids; alignment including 356 taxa], *fliP* [flagellar biosynthetic precursor; encoding a protein of *ca.* 250 amino acids; alignment including 373 taxa], and *flhA* [flagellum biosynthesis protein; encoding a protein of *ca.* 700 amino acids; alignment including 533 taxa], were used as representative genes for phylogenetic reconstruction of Cluster 3. These phylogenies are summarized in **Fig. 4C** and accompany this work as supplementary data in nexus format (**File S1**). For all representative flagellar sequences analyzed, roughly 20 to 25% of homologs detected in the non-redundant database belonged to environmental genomes belonging to the *Chloroflexota*, as determined through their classification in the GTDB release 202. This finding was surprising, as this indicated a large distribution of flagellar structural genes in numerous *Chloroflexota* classes (most notably the *Anaerolineae*, *Chloroflexia*, FW602-bin22, UBA2235, and UBA6077).

***Supplementary Note 6: Cultivation experiments and genomics***

The physiological and biochemical characteristics of strains G233^T^ and YIM 72310^T^ were investigated using routine cultivation on R2A at 60 ^o^C. Growth was also evaluated on *Thermus* 162 medium (DSMZ Medium 878) and T5 agar medium (Yu et al., 2013) at 60 °C. The Gram staining reaction, motility testing, catalase and oxidase activity, hydrolysis of starch, cellulose, Tweens 20, 40, 60 and 80, milk peptonization and coagulation, and reduction of nitrate, and H_2_S production were tested as described previously with R2A as the base medium (Gonzalez et al., 1978; Zhou et al., 2014; Yu et al., 2015). Growth at different temperatures (30-80 ^o^C) was tested on R2A medium by incubating cultures for 15 days. NaCl tolerance growth tests were examined at different NaCl concentrations (0, 0.5, 1, 2, 3, and 4 % w/v) using R2A as a base medium at 60 ^o^C. The pH range for growth was tested at 60 ^o^C for 15 days by culturing the strain in R2A broth, using buffers (15 mM) near their pKa values: pH 4, 5, and 6 (MES), 7 and 8 (HEPES), 9 and 10 (TRIS).

The temperature ranges for growth of YIM 72310^T^ and G233^T^ were slightly higher than T*. bonchosmolovskayae* (Kochetkova et al., 2020), but in a similar range of 45-65 °C, with an optimum at 55-60 °C. Similarly, the pH range for growth of both isolates was 6-8, with an optimum at pH 7, comparable to *T. bonchosmolovskayae* (Kochetkova et al., 2020). Growth was observed at 0-1.0% w/v NaCl concentration. Like *T. bonchosmolovskayae* (Kochetkova et al., 2020), colonies of both new strains were small, circular, and convex. Colonies of the new strains turned pale yellow on all test media after 3 days. *T. bonchosmolovskayae* colonies were described as transparent, but a yellow pigment was detected in cell lysates and polar lipid extracts (Kochetkova et al., 2020). The pigment in *T. bonchosmolovskayae* was speculated to be a carotenoid, which is consistent with a conserved carotenoid biosynthetic cluster.

Routine physiological characterization was done using BIOLOG GEN III, API ZYM, and API 20NE panels, and hydrolysis of polysorbate was tested as described in Gonzalez et al., 1978. Both novel strains were positive for oxidase and catalase. Nitrate reduction, milk coagulation and peptonization, starch hydrolysis, cellulose hydrolysis, and H_2_S production were negative. Tweens 40 and 60 were hydrolyzed, but Tweens 20 and 80 were not.

Three additional approaches were taken to test the use of carbon compounds in triplicate on 10 ml of medium in Balch tubes sealed with air-tight aluminum seals with silicone septa based on genomic predictions and exometabolomic observations. First, sole carbon source tests were performed by the addition of potassium acetate, sodium fumarate, sodium succinate, dextrin, D-ribose, maltose, citric acid, sucrose, potato starch, malic acid, melibiose, oxaloacetic acid, propionic acid, raffinose, casamino acids, yeast extract, proteose peptone and lignin at 0.05% w/v to modified 2R2AW excluding all carbon-containing compounds. Second, growth stimulation tests were performed through the addition of p-coumaric acid, vanillin, azelaic acid, syringic acid, cis-jasmone, sucrose, D-xylose, D-ribose, D-fructose, D-galactose, potassium acetate, sodium pyruvate, sodium succinate, dextrin, maltose, citric acid, potato starch, malic acid, melibiose, oxaloacetic acid, propionic acid, raffinose, mannitol and lignin at 0.05% w/v to 2R2AW. Third, medium drop-out tests were performed to determine utilization of carbon sources within 2R2AW by the exclusion of one of the following ingredients from 2R2AW, specifically casamino acids, dextrose, potato starch, proteose peptone and yeast extract. Microaerophilic and anaerobic growth was tested by the addition of filtered air at O_2_ concentrations of 0, 5, 7 and 11% (vol/vol in headspace) to anoxic 2R2AW, prepared by sparging for 30 minutes with N_2_ gas. To test use of arsenate and nitrite as potential terminal electron acceptors for anaerobic growth, as predicted by genome analysis, arsenate and nitrite were tested at 5 mM in anoxic 2R2AW under an N_2_ atmosphere. Tungstate stimulation tests, predicted by genomic analysis, were performed by the addition of sodium tungstate to W concentrations of 1, 10, and 100 µM in 2R2AW. For all experiments described here, cultures were inoculated in triplicate with exponential-phase cultures at 2% v/v and growth was estimated on days 2, 4, 6, 8, and 10 by measuring the optical density of the broth with the use of a Milton Roy Company Spectronic 20D spectrophotometer at 600 nm wavelength (OD_600_) after incubation at 58.5 °C. Statistically significant growth, or lack of growth, were determined with unpaired t-tests. Shortly, for growth stimulation and medium ingredient drop-out experiments, treatments were compared to positive 2R2AW controls, while sole carbon source experiments entailed comparison of treatments to modified 2R2AW controls containing no carbon sources. For microaerobic growth, lowered concentrations of O_2_ were compared to positive 2R2AW controls with full air, while anaerobic growth with different potential terminal electron acceptors were compared to growth under fully anaerobic conditions.

Medium drop-out of yeast extract significantly reduced growth of both strains (**Table S8**), whereas drop-out of casamino acids only reduced growth of YIM 72310^T^. In contrast, drop-out of potato starch, dextrose, and proteose peptone had no effect on growth. Overall, this is in agreement with exometabolomics experiments, showing no changes in extracellular carbohydrate concentrations during growth of both YIM 72310^T^ and G233^T^ on R2A, and with *T. bonchosmolovskayae* growth on yeast extract; however, *T. bonchosmolovskayae* was also able to grow poorly on starch and several other polysaccharides (Kochetkova et al., 2020).

Microaerobic and anaerobic growth in the absence of terminal electron acceptors were assessed in 2R2AW medium with an N_2_ headspace supplemented with oxygen at 0, 5, 7, 11 and 20% (**Table S6**). Both strains grew under fully aerobic conditions (**Table S6**), but significant growth was also recorded down to 5% headspace O_2_ concentration, consistent with the presence of both high- and low-affinity cytochrome oxidase systems. Neither could grow anaerobically, suggesting fermentation is not possible, similar to *T. bonchosmolovskayae* (Kochetkova et al., 2020) (**Table S6**). No growth was observed with nitrate, nitrite, or arsenate as terminal electron acceptors (**Table S6**).

To determine sensitivity to peptidoglycan-targeting β-lactam antibiotics, 10 ml 2R2AW in Balch tubes was supplemented in triplicate with ampicillin or carbenicillin at concentrations of 0, 1, 2, 4, 8, 16, or 32 μg/ml. The broths were inoculated with YIM 72310 as described before and incubated at 58.5 °C for eight days. Susceptibility was determined by measuring the OD_600_ of each culture compared against controls containing 0 μg/ml of either antibiotic.

To determine whether catechol inhibited growth of YIM 72310^T^, 10 ml 2R2AW in Balch tubes were infused in triplicate with catechol at concentrations of 0, 0.004, 0.008, 0.016, and 0.032% w/v. The broths were inoculated with YIM 72310 and grown as before for eight days. Sensitivity to catechol at varying concentrations was assessed by the OD_600_ of the cultures compared to the control.

***Supplementary Note 7: ^13^C stable isotope experiments***

Following incubation of YIM 72310^T^ on the ^13^C-labeled substrates, sampling of the headspace was done by adding one headspace volume (15 ml) of N_2_ gas to each Balch tube and inverting the tube 10 times to mix. Of the mixed gas, 15 ml was removed from the headspace and transferred to sealed serum bottles containing 15 ml N_2_ gas. From here, headspace gas was collected for cavity ring-down spectroscopy using a Picarro G2201 -i Isotopic Analyzer (Picarro Inc., Santa Clara, CA, USA). Vials were initially autoclaved and filled with one atmosphere of nitrogen gas prior to 5 ml of gaseous sample headspace addition. Each vial then had 5ml of gaseous headspace removed using a gastight syringe injected into the Small Sample Introduction Module 2. The factory protocol was then run with a zero-air dilution and analyzed for ^13^CO_2_/^12^CO_2_ as well as ^13^CH_4_/^12^CH_4_. Ambient air was used between every third injection as a reference point for calibration and to ensure lack of instrument drift.

Nanometer-scale secondary ion mass spectrometry (nanoSIMS) was used to measure assimilation of ^13^C-labeled substrates. After incubation of YIM 72310^T^, cells were cooled on ice and pelleted by centrifugation at 10,000 x g for 5 minutes at 4 °C. The pellet was washed once with 1x phosphate-buffered saline at pH 7.2 and centrifuged at 10,000 x g for 5 minutes. The resulting pellet was resuspended in 500 µl of ice-cold 2% paraformaldehyde and incubated at room temperature for 20 minutes. Cells were centrifuged again at 10,000 x g for 5 minutes and the paraformaldehyde supernatant was decanted. The pellet was washed once using 500 µl ultrapure water and mixed. Cells were pelleted once more by centrifugation at 10,000 x g for 5 minutes and resuspended in 100 µl 50 % ethanol. From here, assimilation of substrates into microbial biomass was analyzed, as ^13^C isotope incorporation was measured in individual cells using the CAMECA NanoSIMS 50 at Lawrence Livermore National Laboratory. After cells were incubated with isotopically labeled substrates, fixed with paraformaldehyde, and washed in double distilled water, 1 µl aliquots of fixed cells were mounted onto silicon wafers and air dried. The silicon wafers were then gold coated to increase surface conductivity. Gold coated cells on a silicon wafer were loaded into the NanoSIMS and scanned for isotopic composition with a 1.5 pA cesium beam. Sputtering equilibrium at each analysis area was achieved with an initial beam current of 90 pA to a depth of ~60 nm to achieve sputtering equilibrium. Five masses (^12^C_2_^−^, ^12^C^13^C^−^, ^12^C^14^N^−^, and ^12^C^15^N^−^, ^32^S^-^) were monitored using electron multipliers at ~7,000 mass resolving power [1.5× correction, per (Pett-Ridge and Weber, 2012)].

Each location targeted by the nanoSIMS had a raster size of 25µm x 25µm and was scanned for 25 cycles which were then composited into a single mosaic image using L’Image software (developed by L Nittler, Carnegie Institution of Washington, Washington, DC, USA). Cells were identified in the mosaic images using an automated particle-finder function based on the ^12^C^14^N^-^ image to extract the isotope ratios. Biomass assimilation of ^13^C-labeled substrates was calculated using the fraction of ^13^C measured in treatment cells (*f_13Cf_*), unlabeled control cells for the background isotope ratio (*f_13Ci_*) and total pool of the ^13^C-labeled substrate found in the media, including dilution by background concentrations of the natural compound (*f_13Cs_*; Hayes 2004; Legin et al., 2014).

$$C_{net\%}= \frac{f_{{13C}_{f}}-f_{{13C}_{i}}}{f_{13C_{s}}-f_{{13C}_{i}}} \times100\%$$

Killed controls of ^13^C-labeled lignin and catechol were included by growing YIM 72310^T^ in 2R2AW containing no labeled substrate for eight days, then fixed with paraformaldehyde. The killed cells were incubated an additional day with labelled lignin and catechol, then centrifuged and resuspended as before. Headspace samples and fixed cells were sent to Lawrence Livermore National Laboratory, Livermore, CA, USA for analysis using cavity ring-down spectroscopy and nanoSIMS, as described above.

Statistical tests were performed using RStudio (RStudio Team, Boston, MA). Treatments were tested against the control group initially using Kruskal-Wallis (Kruskal and Wallis, 1952) tests and followed-up by Dunn Tests (Dunn, 1964) to determine individual treatments statistical significance compared to the control.

The measurements of ^13^CO_2_ indicated that incubations with pyruvate (both C_1_ and C_2/3_ labeled), ribose, acetate, and vanillate were statistically more enriched than the no-addition controls, indicative of respiration of these substrates (**Fig. 6B**). Algal amino acids incubations were also higher than the control, though were not statistically significant. The corresponding nanoSIMS data, which measured the incorporation of substrate carbon into the YIM 72310^T^ cells, showed more significant enrichment following growth on the different substrates than the no-addition control: amino acids, xylose, galactose, ribose, pyruvate, acetate, hemicellulose, and cellulose. Interestingly, vanillate ^13^C, which was detected in the respired carbon, was not found to be incorporated by the cells. On the other hand, cellulose, hemicellulose, amino acids, xylose, and galactose were measured to be incorporated in cells, however, ^13^CO_2_ production were not above the detection limit.

Our incubations of strain YIM 72310^T^ with stable isotope labeled substrates documented in Figure 6 of the main manuscript also included ^13^C labeled lignin, and these cells exhibited extremely high ^13^C enrichment, higher than all the other substrates (data not shown). We suspected that this was caused by an artifact of the labeled lignin sticking to the cells, thus we repeated these incubations, this time with a killed control, as well as incubations (both live and killed) with ^13^C labeled catechol, a smaller molecule that is a major component of lignin. Killed control incubations showed highly labeled cells (**Fig. S5**), but less labeled than the live cells that were incubated with the labeled substrates for a longer period. Based on these data, we cannot rule out that the longer live incubations led to the substrates binding to the cells more efficiently than in the shorter incubations with the killed cells. In addition, based on the images of cells incubated with catechol and lignin, it appeared that hotspots with high ^13^C enrichment were associated with particles that were not shaped like cells. Thus, we cannot determine if lignin and catechol were truly incorporated by the cells for biomass. However, the respiration data suggest that some of the lignin and catechol C was respired, based on higher ^13^CO_2_ in the headspace of incubations with live cells compared to dead cells. We include these inconclusive results here to warn readers about the use of isotope labeled lignin and catechol, which appear to strongly bind to organic material. More experiments will be needed to determine if strain YIM 72310^T^ can metabolize and incorporate lignin and catechol.

**References**

Aramaki, T., Blanc-Mathieu, R., Endo, H., Ohkubo, K., Kanehisa, M., Goto, S., et al. (2020). KofamKOALA: KEGG Ortholog assignment based on profile HMM and adaptive score threshold. *Bioinformatics* 36(7)**,** 2251-2252. doi: 10.1093/bioinformatics/btz859.

Arkin, A.P., Cottingham, R.W., Henry, C.S., Harris, N.L., Stevens, R.L., Maslov, S., et al. (2018). KBase: The United States Department of Energy Systems Biology Knowledgebase. *Nat Biotechnol* 36(7)**,** 566-569. doi: 10.1038/nbt.4163.

Bokulich, N.A., Kaehler, B.D., Rideout, J.R., Dillon, M., Bolyen, E., Knight, R., et al. (2018). Optimizing taxonomic classification of marker-gene amplicon sequences with QIIME 2’s q2-feature-classifier plugin. *Microbiome* 6(1)**,** 90. doi: 10.1186/s40168-018-0470-z.

Bolyen, E., Rideout, J.R., Dillon, M.R., Bokulich, N.A., Abnet, C.C., Al-Ghalith, G.A., et al. (2019). Reproducible, interactive, scalable and extensible microbiome data science using QIIME 2. *Nat Biotechnol* 37(8)**,** 852-857. doi: 10.1038/s41587-019-0209-9.

Brettin, T., Davis, J.J., Disz, T., Edwards, R.A., Gerdes, S., Olsen, G.J., et al. (2015). RASTtk: A modular and extensible implementation of the RAST algorithm for building custom annotation pipelines and annotating batches of genomes. *Sci Rep* 5(1)**,** 8365. doi: 10.1038/srep08365.

Buckel, W., Kung, J.W., Boll, M. (2014) The benzoyl-coenzyme A reductase and 2-hydroxyacyl-coenzyme A dehydratase radical enzyme family. *ChemBioChem* 15, 2188-2194. doi: 10.1002/cbic.201402270.

Camacho, C., Coulouris, G., Avagyan, V., Ma, N., Papadopoulos, J., Bealer, K., et al. (2009). BLAST+: architecture and applications. *BMC Bioinformatics* 10(1)**,** 421. doi: 10.1186/1471-2105-10-421.

Cantalapiedra, C.P., Hernández-Plaza, A., Letunic, I., Bork, P., and Huerta-Cepas, J. (2021). eggNOG-mapper v2: Functional Annotation, Orthology Assignments, and Domain Prediction at the Metagenomic Scale. *Mol Biol Evol* 38(12)**,** 5825-5829. doi: 10.1093/molbev/msab293.

Chernomor, O., von Haeseler, A., and Minh, B.Q. (2016). Terrace aware data structure forphylogenomic inference from supermatrices. *Syst Biol* 65(6)**,** 997-1008. doi: 10.1093/sysbio/syw037.

Cole, J.K., Gieler, B.A., Heisler, D.L., Palisoc, M.M., Williams, A.J., Dohnalkova, A.C., et al. (2013). *Kallotenue papyrolyticum* gen. nov., sp. nov., a cellulolytic and filamentous thermophile that represents a novel lineage (*Kallotenuales* ord. nov., *Kallotenuaceae* fam. nov.) within the class *Chloroflexia*. *Int J Syst Evol Microbiol* 63**,** 4675-4682. doi: 10.1099/ijs.0.053348-0.

Costa, K.C., Navarro, J.B., Shock, E.L., Zhang, C.L., Soukup, D., and Hedlund, B.P. (2009). Microbiology and geochemistry of great boiling and mud hot springs in the United States Great Basin. *Extremophiles* 13(3)**,** 447-459. doi: 10.1007/s00792-009-0230-x.

Csűös, M. (2010). Count: evolutionary analysis of phylogenetic profiles with parsimony and likelihood. *Bioinformatics* 26(15)**,** 1910-1912. doi: 10.1093/bioinformatics/btq315.

Darriba, D., Taboada, G.L., Doallo, R., and Posada, D. (2011). ProtTest 3: fast selection of best-fit models of protein evolution. *Bioinformatics* 27(8)**,** 1164-1165. doi: 10.1093/bioinformatics/btr088.

Dodsworth, J.A., Gevorkian, J., Despujos, F., Cole, J.K., Murugapiran, S.K., Ming, H., et al. (2014). *Thermoflexus hugenholtzii* gen. nov., sp. nov., a thermophilic, microaerophilic, filamentous bacterium representing a novel class in the *Chloroflexi,* *Thermoflexia* classis nov., and description of *Thermoflexaceae* fam. nov. and *Thermoflexales* ord. nov. *Int J Syst Evol Microbiol* 64**,** 2119-2127. doi: 10.1099/ijs.0.055855-0.

Dunn, O.J. (1964). Multiple comparisons using rank sums. *Technometrics* 6(3)**,** 241-252. doi: 10.1080/00401706.1964.10490181.

Gonzalez, C., Gutierrez, C., and Ramirez, C. (1978). *Halobacterium vallismorti*s sp. nov. An amylolytic and carbohydrate-metabolizing, extremely halophilic bacterium. *Canadian Journal of Microbiology* 24(6)**,** 710-715. doi: 10.1139/m78-119.

Guindon, S., Dufayard, J.-F., Lefort, V., Anisimova, M., Hordijk, W., and Gascuel, O. (2010). New algorithms and methods to estimate maximum-likelihood phylogenies: assessing the performance of PhyML 3.0. *Systematic Biology* 59(3)**,** 307-321. doi: 10.1093/sysbio/syq010.

Guo, Q., Planer-Friedrich, B., Liu, M., Li, J., Zhou, C., and Wang, Y. (2017). Arsenic and thioarsenic species in the hot springs of the Rehai magmatic geothermal system, Tengchong volcanic region, China. *Chemical Geology* 453**,** 12-20. doi: https://doi.org/10.1016/j.chemgeo.2017.02.010.

Hayes, J. (2004) An Introduction to Isotope Calculations, Woods Hole Oceanographic Institution, https://www.whoi.edu/cms/files/jhayes/2005/9/IsoCalcs30Sept04_5183.pdf

Hoang, D.T., Chernomor, O., von Haeseler, A., Minh, B.Q., and Vinh, L.S. (2018). UFBoot2: Improving the ultrafast bootstrap approximation. *Molecular Biology and Evolution* 35(2)**,** 518-522. doi: 10.1093/molbev/msx281.

Hyatt, D., Chen, G.-L., LoCascio, P.F., Land, M.L., Larimer, F.W., and Hauser, L.J. (2010). Prodigal: prokaryotic gene recognition and translation initiation site identification. *BMC Bioinformatics* 11(1)**,** 119. doi: 10.1186/1471-2105-11-119.

Kalyaanamoorthy, S., Minh, B.Q., Wong, T.K.F., von Haeseler, A., and Jermiin, L.S. (2017). ModelFinder: fast model selection for accurate phylogenetic estimates. *Nature Methods* 14(6)**,** 587-589. doi: 10.1038/nmeth.4285.

Katoh, K., and Standley, D.M. (2013). MAFFT Multiple Sequence Alignment Software Version 7: Improvements in Performance and Usability. *Molecular Biology and Evolution* 30(4)**,** 772-780. doi: 10.1093/molbev/mst010.

Kochetkova, T.V., Zayulina, K.S., Zhigarkov, V.S., Minaev, N.V., Chichkov, B.N., Novikov, A.A., et al. (2020). *Tepidiforma bonchosmolovskayae* gen. nov., sp. nov., a moderately thermophilic *Chloroflexi* bacterium from a Chukotka hot spring (Arctic, Russia), representing a novel class, *Tepidiformia*, which includes the previously uncultivated lineage OLB14. *Int J Syst Evol Microbiol* 70(2)**,** 1192-1202. doi: 10.1099/ijsem.0.003902.

Kruskal, W.H., and Wallis, W.A. (1952). Use of ranks in one-criterion variance analysis. *Journal of the American Statistical Association* 47(260)**,** 583-621. doi: 10.2307/2280779.

Kück, P., and Longo, G.C. (2014). FASconCAT-G: extensive functions for multiple sequence alignment preparations concerning phylogenetic studies. *Frontiers in Zoology* 11(1)**,** 81. doi: 10.1186/s12983-014-0081-x.

Kumar, S., Stecher, G., Li, M., Knyaz, C., and Tamura, K. (2018). MEGA X: molecular evolutionary genetics analysis across computing platforms. *Molecular Biology and Evolution* 35(6)**,** 1547-1549. doi: 10.1093/molbev/msy096.

Letunic, I., and Bork, P. (2021). Interactive Tree Of Life (iTOL) v5: an online tool for phylogenetic tree display and annotation. *Nucleic Acids Research* 49(W1)**,** W293-W296. doi: 10.1093/nar/gkab301.

Li, L., Stoeckert, C.J., and Roos, D.S. (2003). OrthoMCL: identification of ortholog groups for eukaryotic genomes. *Genome Research* 13(9)**,** 2178-2189.

McDonald, D., Clemente, J.C., Kuczynski, J., Rideout, J.R., Stombaugh, J., Wendel, D., et al. (2012). The Biological Observation Matrix (BIOM) format or: how I learned to stop worrying and love the ome-ome. *GigaScience* 1(1)**,** 2047-2217X-2041-2047. doi: 10.1186/2047-217X-1-7.

Nguyen, L.-T., Schmidt, H.A., von Haeseler, A., and Minh, B.Q. (2015). IQ-TREE: A fast and effective stochastic algorithm for estimating maximum-lkelihood phylogenies. *Molecular Biology and Evolution* 32(1)**,** 268-274. doi: 10.1093/molbev/msu300.

Overbeek, R., Olson, R., Pusch, G.D., Olsen, G.J., Davis, J.J., Disz, T., et al. (2014). The SEED and the Rapid Annotation of microbial genomes using Subsystems Technology (RAST). *Nucleic Acids Research* 42(D1)**,** D206-D214. doi: 10.1093/nar/gkt1226.

Pett-Ridge, J., and Weber, P.K. (2012). "NanoSIP: NanoSIMS applications for microbial biology," in *Microbial Systems Biology: Methods and Protocols,* ed. A. Navid. (Totowa, NJ: Humana Press), 375-408.

Rodriguez-R, L., and Konstantinidis, K. (2016). "The enveomics collection: a toolbox for specialized analyses of microbial genomes and metagenomes". PeerJ Preprints).

Rozewicki, J., Li, S., Amada, K.M., Standley, D.M., and Katoh, K. (2019). MAFFT-DASH: integrated protein sequence and structural alignment. *Nucleic Acids Research* 47(W1)**,** W5-W10. doi: 10.1093/nar/gkz342.

Sorokin, D.Y., Vejmelkova, D., Lücker, S., Streshinskaya, G.M., Rijpstra, W.I.C., Damste, J.S.S., et al. (2014). *Nitrolancea hollandica* gen. nov., sp. nov., a chemolithoautotrophic nitrite-oxidizing bacterium isolated from a bioreactor belonging to the phylum *Chloroflexi*. *Int J Syst Evol Microbiol* 64**,** 1859-1865. doi: 10.1099/ijs.0.062232-0.

Spero, M., A., Brickner, J., R., Mollet, J., T., Pisithkul, T., Amador-Noguez, D., and Donohue Timothy, J. (2016). Different functions of phylogenetically distinct bacterial complex I isozymes. *Journal of Bacteriology* 198(8)**,** 1268-1280. doi: 10.1128/JB.01025-15.

Wang, L., Yan, J., Wise, M.J., Liu, Q., Asenso, J., Huang, Y., et al. (2018). Distribution patterns of polyphosphate metabolism pathway and its relationships with bacterial durability and virulence. *Front Microbiol* 9**,** 782. doi: 10.3389/fmicb.2018.00782.

Yamada, T., Sekiguchi, Y., Hanada, S., Imachi, H., Ohashi, A., Harada, H., et al. (2006). *Anaerolinea thermolimosa* sp. nov., *Levilinea saccharolytica* gen. nov., sp. nov. and *Leptolinea tardivitalis* gen. nov., sp. nov., novel filamentous anaerobes, and description of the new classes *Anaerolineae* classis nov. and *Caldilineae* classis nov. in the bacterial phylum *Chloroflexi*. *I J Syst Evol Microbiol* 56(6)**,** 1331-1340. doi: 10.1099/ijs.0.64169-0.

Yan, G., Chen, X., Du, S., Deng, Z., Wang, L., and Chen, S. (2019). Genetic mechanisms of arsenic detoxification and metabolism in bacteria. *Current Genetics* 65(2)**,** 329-338. doi: 10.1007/s00294-018-0894-9.

Yu, T.-T., Ming, H., Yao, J.-C., Zhou, E.-M., Park, D.-J., Hozzein, W.N., et al. (2015). *Thermus amyloliquefaciens* sp. nov., isolated from a hot spring sediment sample. *International Journal of Systematic and Evolutionary Microbiology* 65(Pt_8)**,** 2491-2495. doi: https://doi.org/10.1099/ijs.0.000289.

Yu, T.-T., Yao, J.-C., Ming, H., Yin, Y.-R., Zhou, E.-M., Liu, M.-J., et al. (2013). *Thermus tengchongensis* sp. nov., isolated from a geothermally heated soil sample in Tengchong, Yunnan, south-west China. *Antonie van Leeuwenhoek* 103(3)**,** 513-518. doi: 10.1007/s10482-012-9833-9.

Zhou, E.-M., Yu, T.-T., Liu, L., Ming, H., Yin, Y.-R., Dong, L., et al. (2014). *Geothermomicrobium terrae* gen. nov., sp. nov., a novel member of the family *Thermoactinomycetaceae*. *International Journal of Systematic and Evolutionary Microbiology* 64(Pt_9)**,** 2998-3004. doi: https://doi.org/10.1099/ijs.0.059766-0.
