# Supplementary material for: Thermophilic Dehalococcoidia with unusual traits shed light on an unexpected past
Source: ISME J. 2023 Apr 11;17(7):952–66. doi: 10.1038/s41396-023-01405-0 (PMC10284905; doi:10.1038/s41396-023-01405-0)
Supplement: Supplementary file 2 — Supplemental Figures [file 41396_2023_1405_MOESM2_ESM.pdf]

Thermophilic *Dehalococcoidia* with unusual traits shed light on an unexpected past

Marike Palmer, Jonathan K. Covington, En-Min Zhou, Scott C. Thomas, Neeli Habib, Cale O. Seymour, Dengxun Lai, Juliet Johnston, Ameena Hashimi, Jian-Yu Jiao, Alise R. Muok, Lan Liu, Wen-Dong Xian, Xiao-Yang Zhi, Meng-Meng Li, Leslie P. Silva, Benjamin P. Bowen, Katherine Louie, Ariane Briegel, Jennifer Pett-Ridge, Peter K. Weber, Elitza I. Tocheva, Tanja Woyke, Trent R. Northen, Xavier Mayali, Wen-Jun Li, Brian P. Hedlund

Supplementary Tables

1. **Table S1.** High-quality *Chloroflexota* genomes used in this study
2. **Table S2.** High-quality *Dehalococcoidia* genomes used in this study
3. **Table S3.** EMP data for the *Tepidiformaceae* and *Tepidiforma*
4. **Table S4.** Prevalence of *Tepidiformaceae* and *Tepidiforma* in EMP datasets
5. **Table S5.** Core genome of the *Tepidiformales*
6. **Table S6.** Genome annotations for YIM 72310^{T}
7. **Table S7.** Genome annotations for G233^{T}
8. **Table S8.** Anaerobic growth experiments
9. **Table S9.** Sole carbon source experiments
10. **Table S10.** Medium drop-out experiments
11. **Table S11.** Growth stimulation experiments
12. **Table S12.** Exometabolomics data for YIM 72310^{T}
13. **Table S13.** Exometabolomics data for G233^{T}
14. **Table S14.** Summary of metabolite identification in negative mode
15. **Table S15.** Summary of compound metadata in negative mode
16. **Table S16.** Summary of metabolite identification in positive mode
17. **Table S17.** Summary of compound metadata in positive mode
18. **Table S18.** CAZymes annotated for YIM 72310^{T}
19. **Table S19.** CAZymes annotated for G233^{T}
20. **Table S20.** Quantitative PCR results for detection of *Tepidiforma* in Great Boiling Spring
21. **Table S21.** MIGS data for YIM 72310^{T} and G233^{T}
22. **Table S22.** Growth at varying tungsten concentrations

Supplementary Datasets

1. **File S1.** Key flagellar and aromatics degradation gene trees
2. **MassIVE dataset 1.** Exometabolomics for *Tepidiforma* spp. grown on R2A broth (https://doi.org/10.25345/C57S7HX50)
3. **FigShare Collection 1.** Exometabolomics analysis for MassIVE dataset MSV000090480 (https://doi.org/10.6084/m9.figshare.c.6236283.v1)

Supplementary Figures

1. **Figure S1.** *Chloroflexota* Bac120 phylogeny
2. **Figure S2.** EMP distribution
3. **Figure S3.** Growth of YIM 72310^T with β-lactam antibiotics
4. **Figure S4.** Exometabolomics data
5. **Figure S5.** Total CO2 produced from labeled and unlabeled substrates
6. **Figure S6.** Lignin $^{13}CO_2$ production and ^{13}C incorporation data
7. **Figure S7.** Aromatics degradation pathways, presence of genes and summary of phylogenies
8. **Figure S8.** Phylogenies of key aromatic degradation pathway proteins

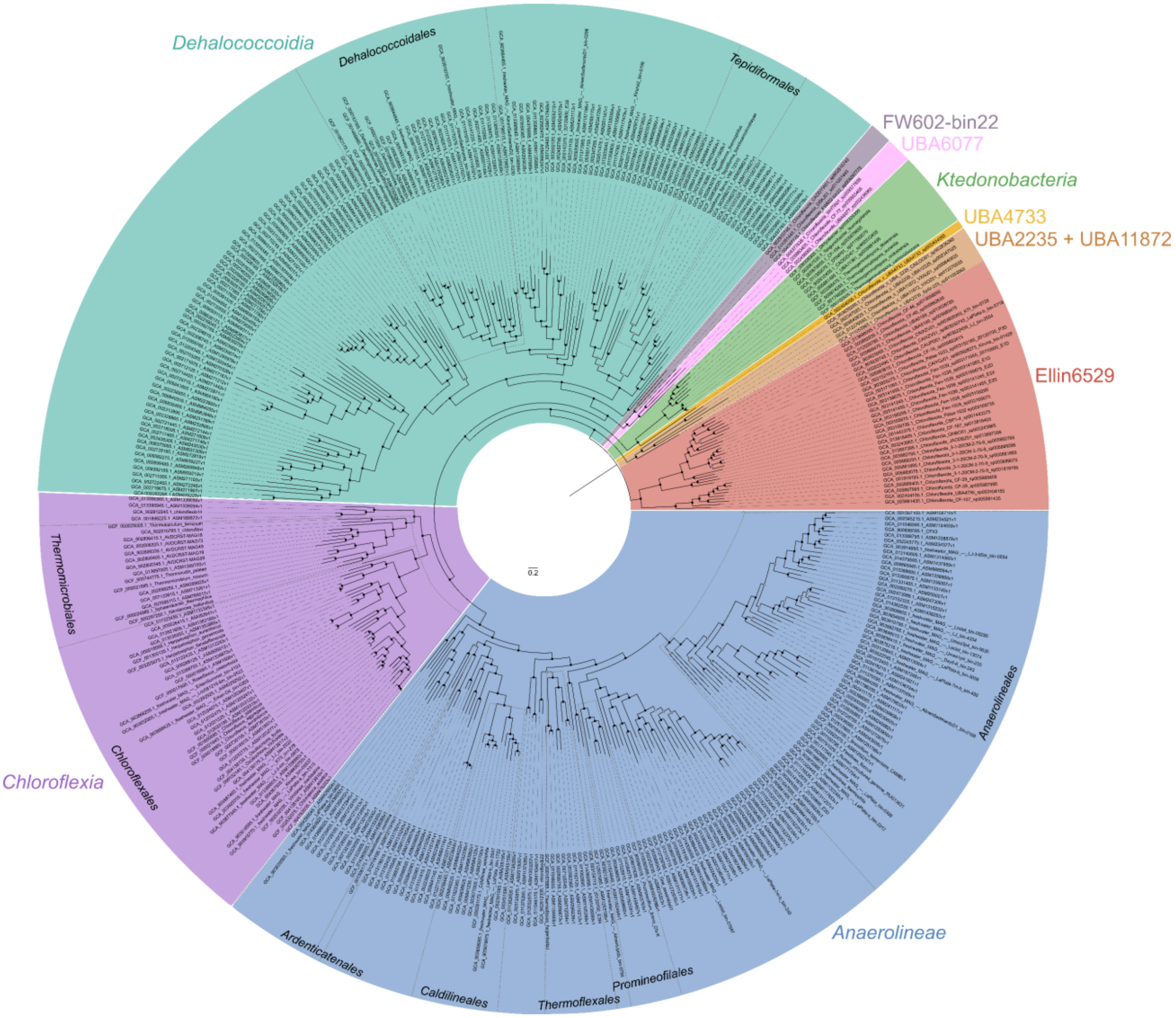

Figure S1. *Chloroflexota* Bac120 phylogeny. An approximate maximum-likelihood phylogeny constructed from the concatenated bacterial markers as extracted and aligned with GTDB-Tk v. 1.4.1, for all high-quality genomes representing species-groups belonging to the phylum *Chloroflexota*. All markers were evaluated to determine the best-fit evolutionary model with ProtTest v. 3.4 and concatenated and partitioned in FAsconCAT-G v. 1.04. Classes as identified in the Genome Taxonomy Database are represented by colored segments, and order-level groups with cultivated representatives within the phylum are indicated as wedges. Branches supported by > 0.95 support from 1,000 replications with Shimodaira-Hasegawa support are indicated as black dots at nodes.

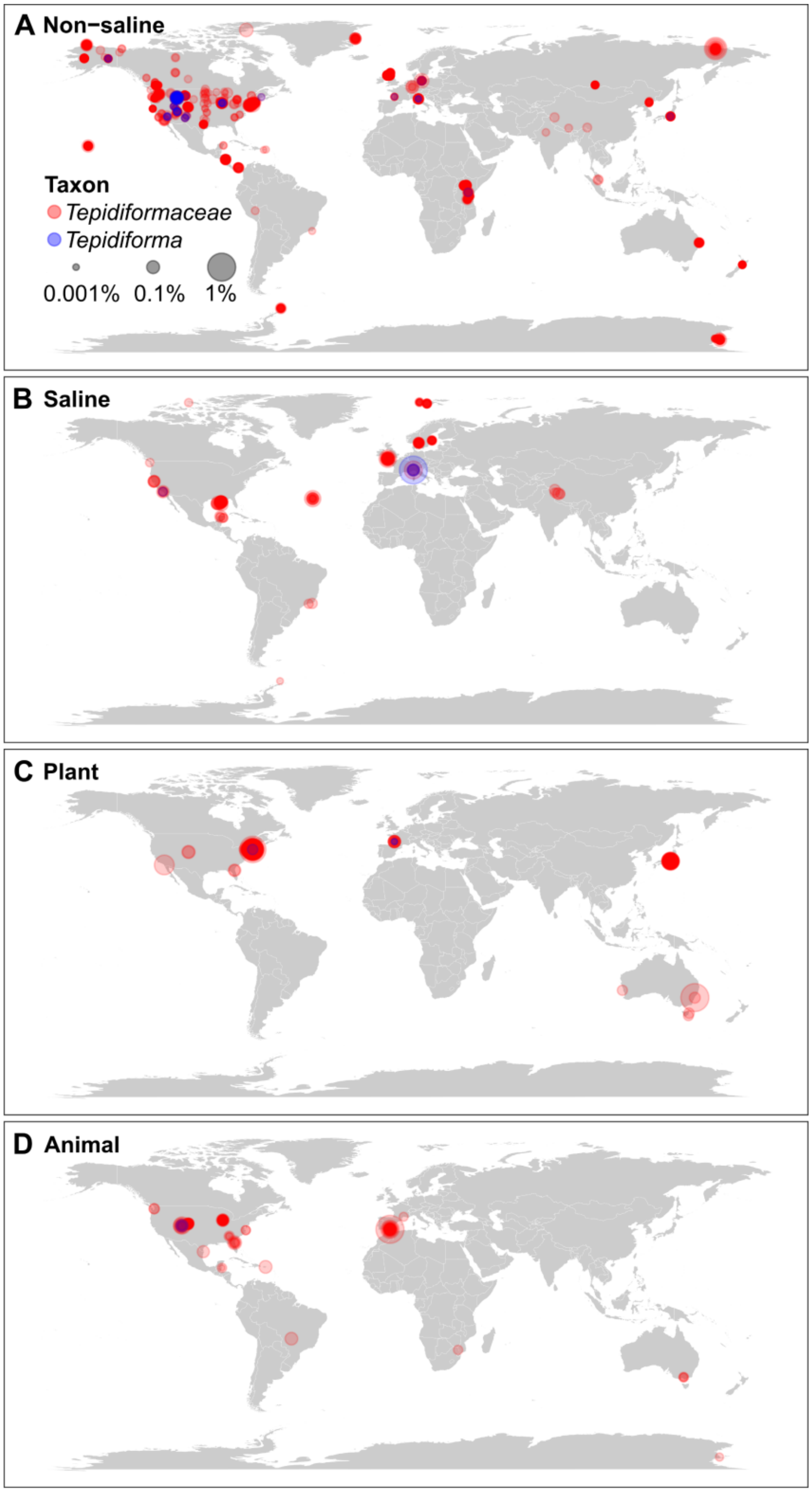

Figure S2. Earth Microbiome Project data for the *Tepidiformaceae* and *Tepidiforma*. Geographic distribution of *Tepidiformaceae* and *Tepidiforma* within the EMP Qiita database (1) across empo-level 2 biomes. Each point represents a microbial community containing the family (red) or genus (blue) based on 16S rRNA gene sequences.

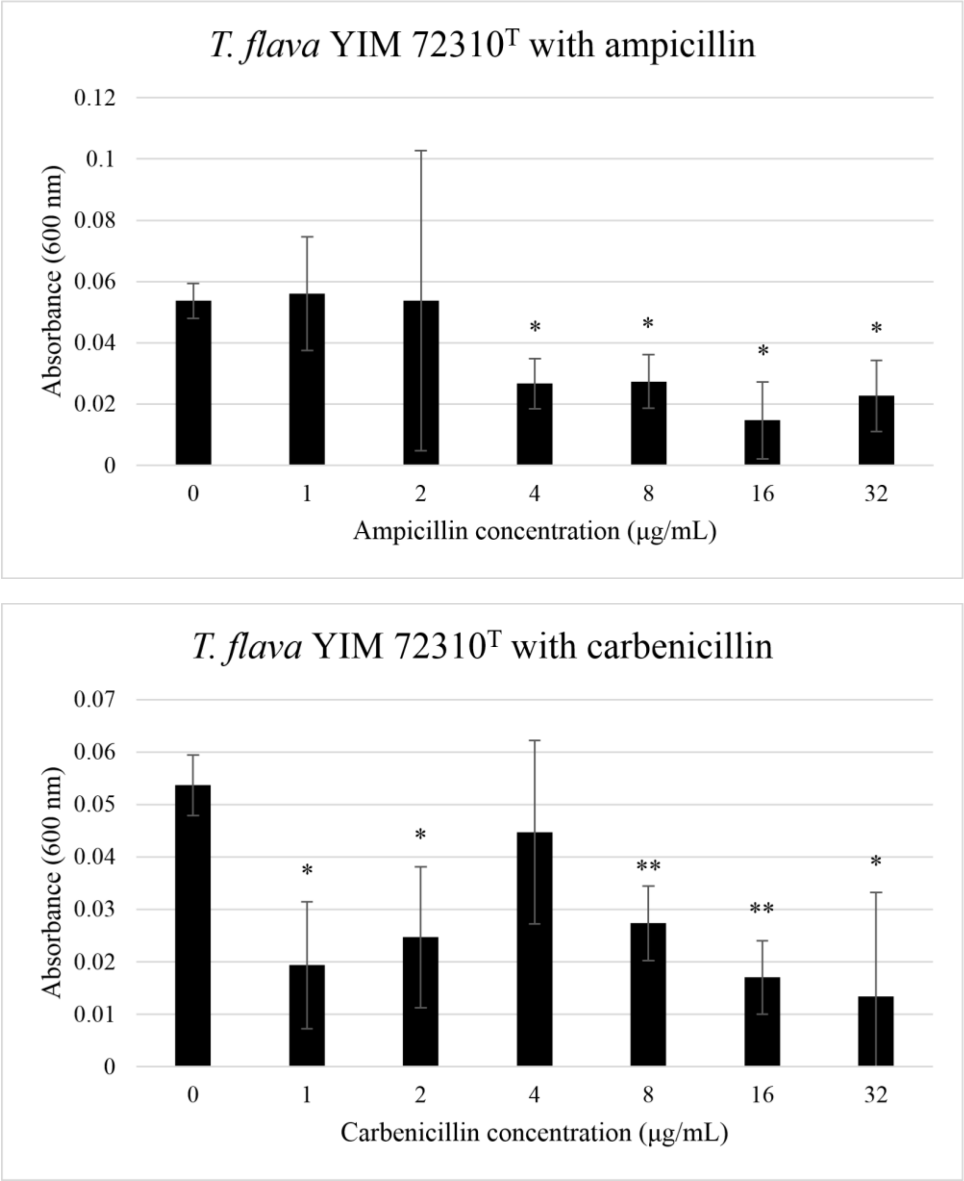

Figure S3. Growth of strain YIM 72310^T on 2R2AW broth containing varying concentrations of β-lactam antibiotics. Effects on growth were assessed using unpaired t-tests and were either significant (p-value < 0.05) or highly significant (p-value < 0.005) compared to the 0 µg/mL controls.

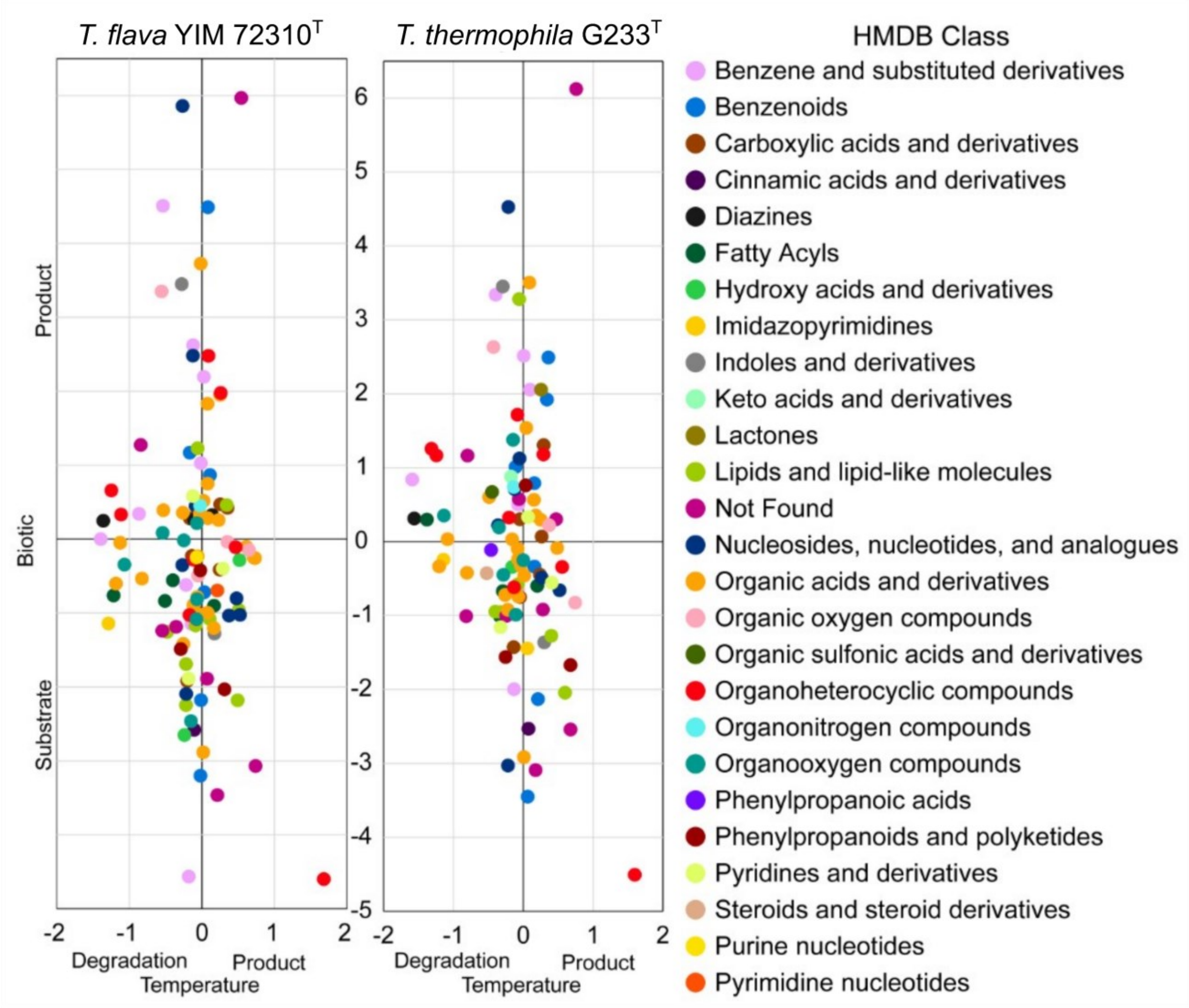

Figure S4. Exometabolomics results demonstrate metabolisms of heterocycles and lipids. Log2 fold changes in peak height for metabolites that differed significantly between treatments (p-value < 0.05 for ANOVA with Tukey's HSD). Colors indicate the Human Metabolome Database (HMDB) metabolite Class for specific compounds. Compounds can be identified in Table S10 and S11 and are ordered vertically as shown on this graph. Compounds with more negative values on the y-axis indicate biological consumption, while positive values on the y-axis indicate biological production. Similarly, negative values on the x-axis indicate thermal degradation, while positive values on the x-axis indicate thermal production.

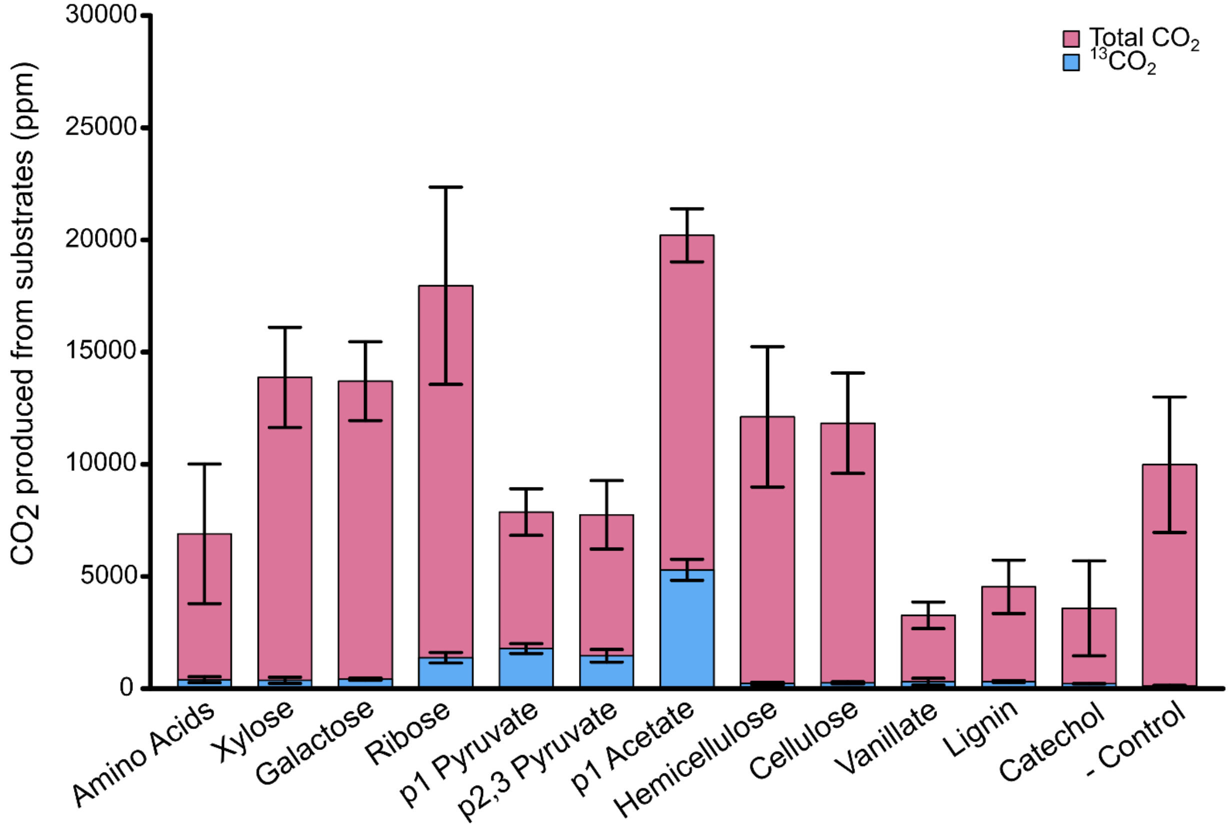

Figure S5. Total CO_2 produced from labeled and unlabeled substrates. CO_2 produced from labeled substrate is indicated in blue (data shown in **Fig. 6B**), while all CO_2 produced from labeled and unlabeled substrate is indicated in pink. The total CO_2 produced from substrates do not differ statistically from the experimental control.

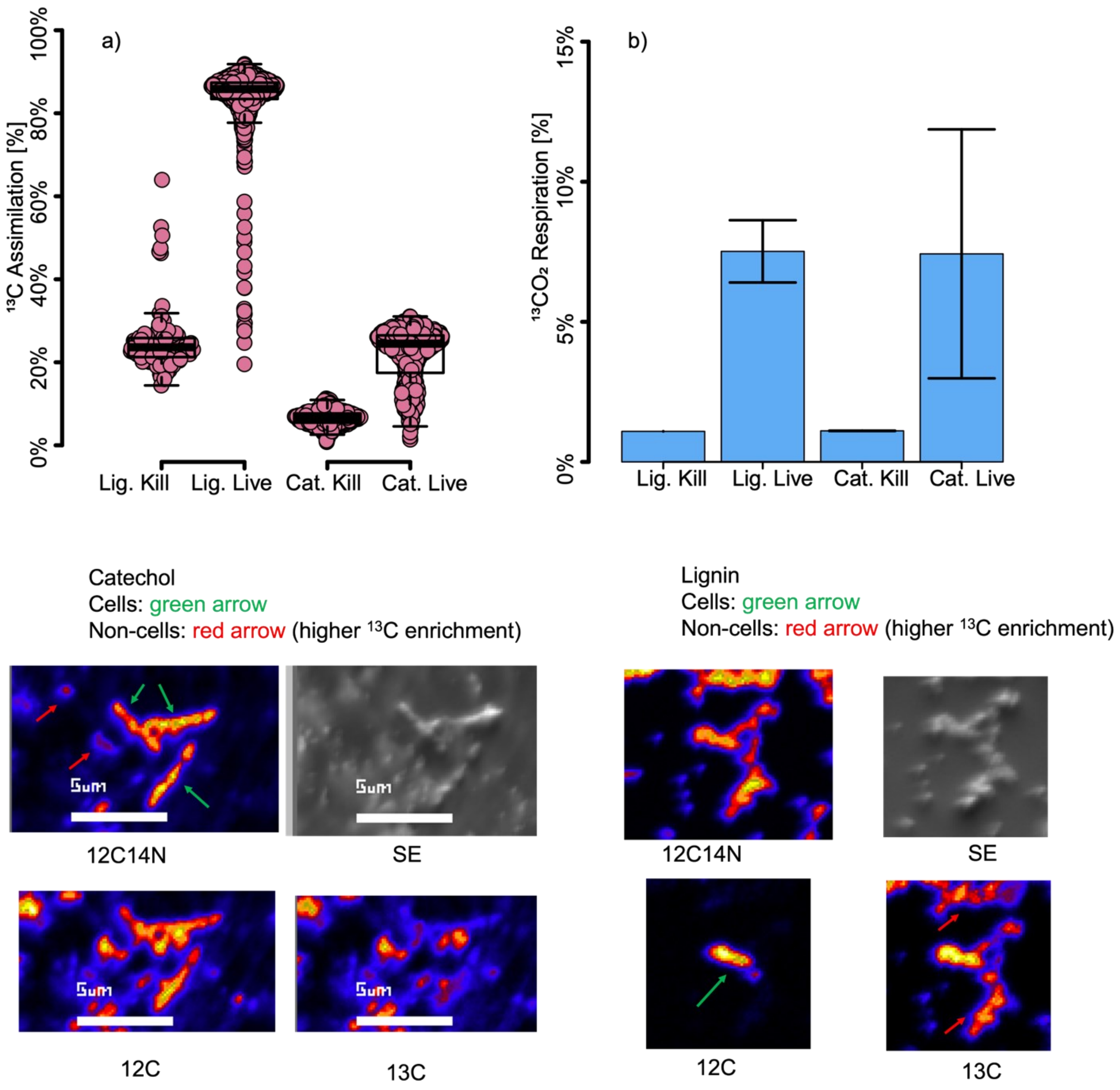

Figure S6. Single cell isotope ^{13}C labeling (a) and $^{13}CO_2$ production (b) of ^{13}C lignin and ^{13}C catechol. While the live cells were more strongly labeled than the killed cells, these data are not definitive for incorporation, as the nanoSIMS images (c, d) suggest the lignin and catechol bind to non-cellular organic material (red arrows).

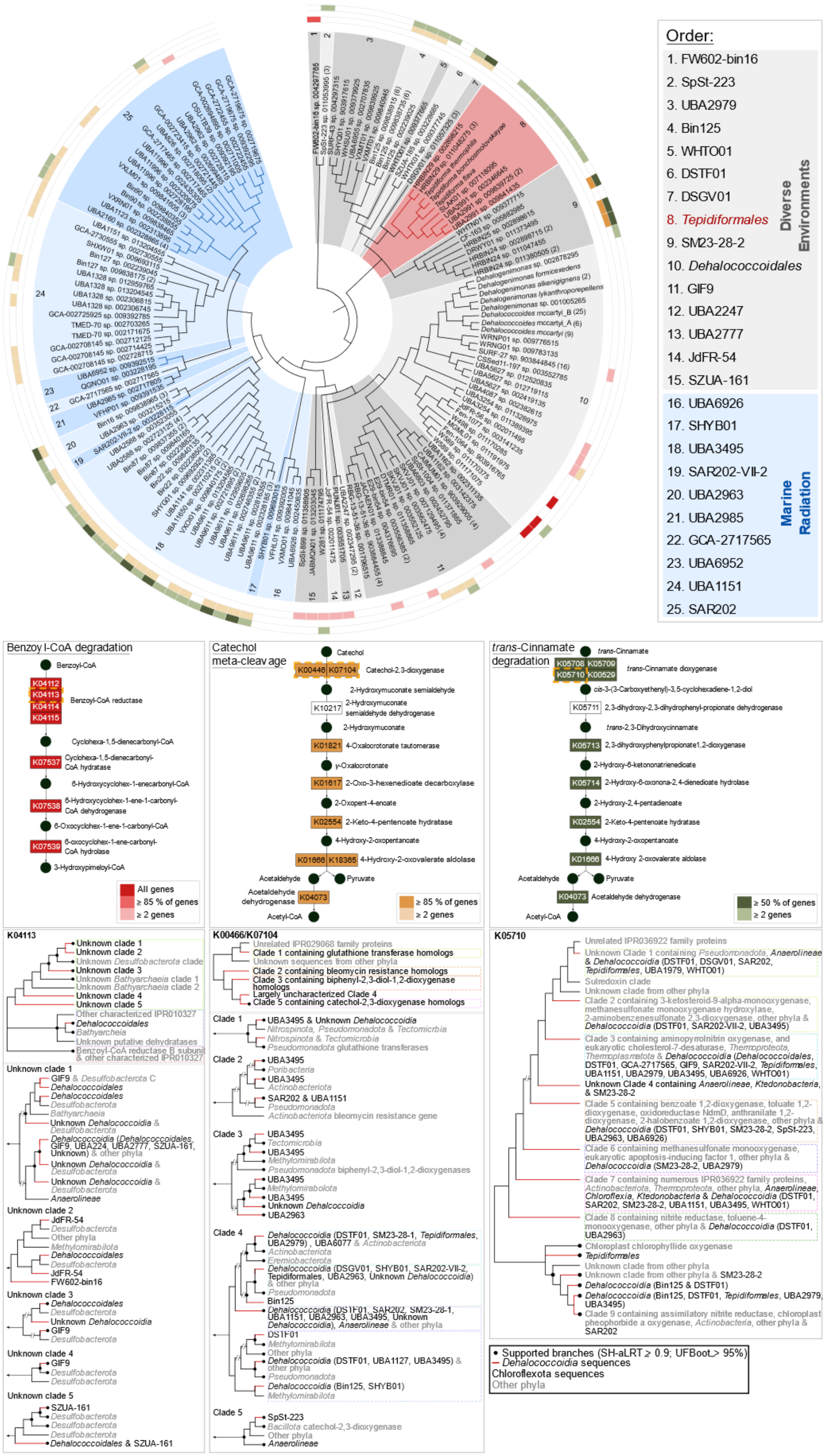
Order:
1. FW602-bin16
2. SpSt-223
3. UBA2979
4. Bin125
5. WHTO01
6. DSTF01
7. DSGV01
8. Tepidiformales
9. SM23-28-2
10. Dehalococcoidales
11. GIF9
12. UBA2247
13. UBA2777
14. JdFR-54
15. SZUA-161
16. UBA6926
17. SHYB01
18. UBA3495
19. SAR202-VII-2
20. UBA2963
21. UBA2985
22. GCA-2717565
23. UBA6952
24. UBA1151
25. SAR202
Diverse Environments
Marine Radiation
Benzoyl-CoA degradation
Catechol meta-cleavage
trans-Cinnamate degradation
All genes
≥ 85 % of genes
≥ 2 genes
≥ 50 % of genes
K04113
K00466/K07104
K05710
Supported branches (SH-aLRT ≥ 0.9; UFBoot > 95%)
Dehalococcoidia sequences
Chloroflexota sequences
Other phyla

Figure S7. Distribution of genes involved in aromatics degradation pathways across the class *Dehalococcoidia*. Order-level groups within the class *Dehalococcoidia* are indicated with alternating shades, with classes from diverse environments indicated in grey and the marine radiation indicated in blue, while indicated numbers correspond to the numbers indicated in the key. Several pathways for the degradation of aromatic compounds are depicted in different colors. Distribution of the genes across the class is colored according to the color of degradation pathways. Simplified cladograms inferred from maximum-likelihood phylogenies of key aromatic degradation proteins (**Fig. S8**), indicated in orange dotted lines in the pathways, are shown below each corresponding KEGG orthology number. Cladograms are inferred from midpoint-rooted phylogenies. Branches with sequences from *Dehalococcoidia* genomes are indicated in red, while all *Chloroflexota* are indicated in black text, and all other phyla are indicated in grey text. Branches supported with ≥ 0.9 SH-aLRT and ≥ 95 % ultrafast bootstrap values are indicated with filled dots.

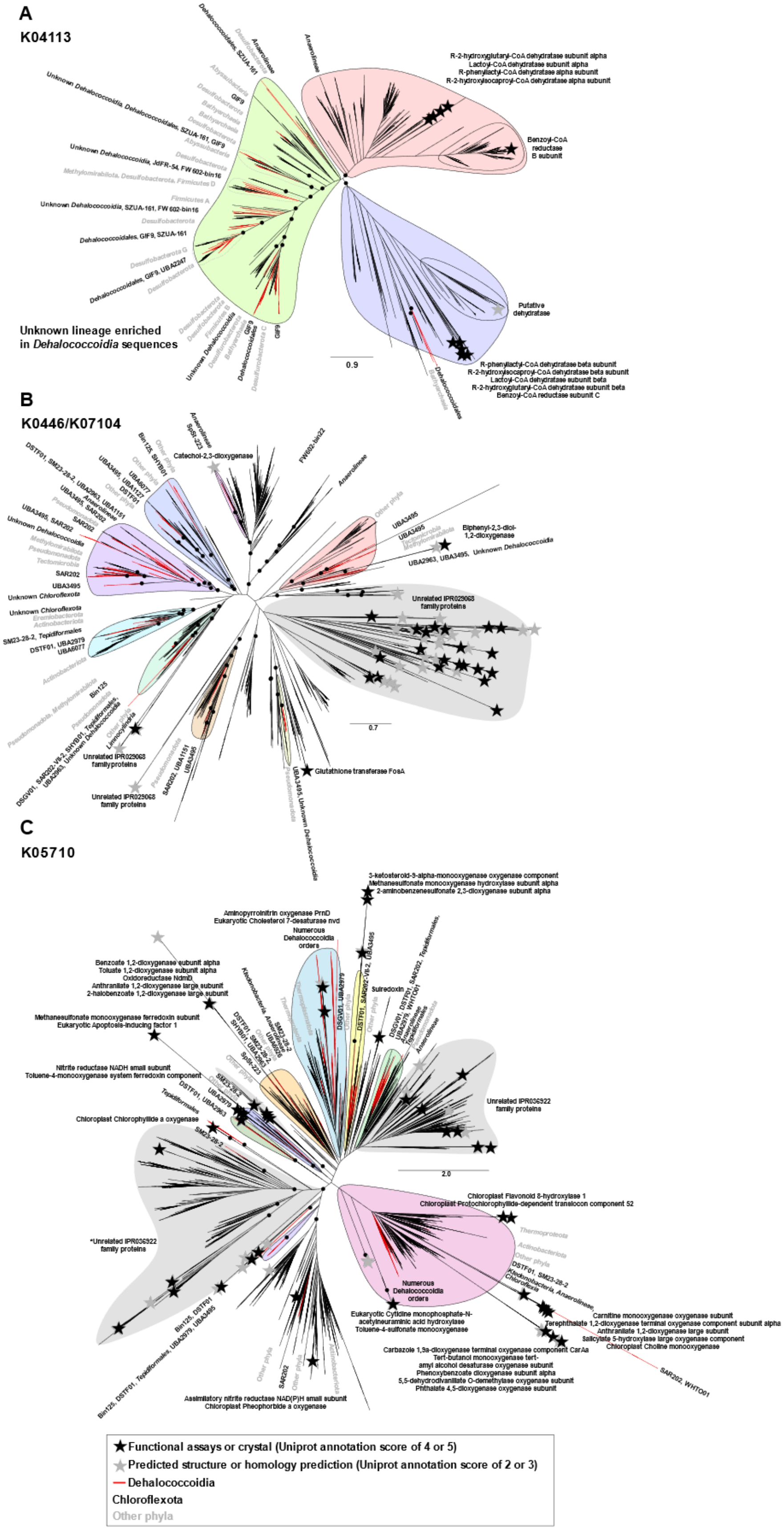
A
K04113
R-2-hydroxyglutaryl-CoA dehydratase subunit alpha
Lactoyl-CoA dehydratase subunit alpha
R-phenyllactyl-CoA dehydratase alpha subunit
R-2-hydroxyisocaproyl-CoA dehydratase alpha subunit
Benzoyl-CoA reductase B subunit
Putative dehydratase
R-phenyllactyl-CoA dehydratase beta subunit
R-2-hydroxyisocaproyl-CoA dehydratase beta subunit
Lactoyl-CoA dehydratase subunit beta
R-2-hydroxyglutaryl-CoA dehydratase subunit beta
Benzoyl-CoA reductase subunit C
Unknown lineage enriched in *Dehalococcoidia* sequences
0.9
B
K0446/K07104
Catechol-2,3-dioxygenase
Biphenyl-2,3-diol-1,2-dioxygenase
Unrelated IPR029068 family proteins
Glutathione transferase FosA
0.7
C
K05710
3-ketosteroid-9-alpha-monooxygenase oxygenase component
Methanesulfonate monooxygenase hydroxylase subunit alpha
2-aminobenzenesulfonate 2,3-dioxygenase subunit alpha
Aminopyrrolnitrin oxygenase PrnD
Eukaryotic Cholesterol 7-desaturase nvd
Numerous Dehalococcoidia orders
Benzoate 1,2-dioxygenase subunit alpha
Toluate 1,2-dioxygenase subunit alpha
Oxidoreductase NdmD
Anthranilate 1,2-dioxygenase large subunit
2-halobenzoate 1,2-dioxygenase large subunit
Methanesulfonate monooxygenase ferredoxin subunit
Eukaryotic Apoptosis-inducing factor 1
Nitrite reductase NADH small subunit
Toluene-4-monooxygenase system ferredoxin component
Chloroplast Chlorophyllide a oxygenase
Sulredoxin
Unrelated IPR036922 family proteins
2.0
Chloroplast Flavonoid 8-hydroxylase 1
Chloroplast Protochlorophyllide-dependent translocon component 52
*Unrelated IPR036922 family proteins
Eukaryotic Cytidine monophosphate-N-acetylneuraminic acid hydroxylase
Toluene-4-sulfonate monooxygenase
Carnitine monooxygenase oxygenase subunit
Terephthalate 1,2-dioxygenase terminal oxygenase component subunit alpha
Anthranilate 1,2-dioxygenase large subunit
Salicylate 5-hydroxylase large oxygenase component
Chloroplast Choline monooxygenase
Carbazole 1,9a-dioxygenase terminal oxygenase component CarAa
Tert-butanol monooxygenase tert-amyl alcohol desaturase oxygenase subunit
Phenoxybenzoate dioxygenase subunit alpha
5,5-dehydrodivanillate O-demethylase oxygenase subunit
Phthalate 4,5-dioxygenase oxygenase subunit
Assimilatory nitrite reductase NAD(P)H small subunit
Chloroplast Pheophorbide a oxygenase
Functional assays or crystal (Uniprot annotation score of 4 or 5)
Predicted structure or homology prediction (Uniprot annotation score of 2 or 3)
Dehalococcoidia
Chloroflexota
Other phyla

Figure S8. Maximum-Likelihood phylogenies of key aromatics degradation proteins. Branches of sequences derived from *Dehalococcoidia* genomes are indicated with red, while all *Chloroflexota* taxon names are indicated in black, and other phyla are indicated with grey. Supported backbone branches (≥ 0.9 SH-aLRT and 95 % ultrafast bootstrap) are indicated with filled dots at nodes. (A) Functionally characterized proteins belonging to the FldB/FldC dehydratase alpha/beta subunit (IPR010327) protein family are indicated with stars. Overall, three supported groups can be recovered with most sequences annotated as the B subunit of benzoyl-CoA reductase in *Dehalococcoidia* genomes grouping in an unknown lineage with no functionally characterized homologs. (B) Functionally characterized proteins of the glyoxalase/bleomycin resistance/dihydroxybiphenyl dioxygenase (IPR029068) protein superfamily are indicated with stars and indicated when grouping close to *Dehalococcoidia* sequences. (C) Functionally characterized proteins of the Rieske [2Fe-2S] iron-sulphur domain (IPR036922) protein superfamily are indicated with stars and indicated when grouping close to *Dehalococcoidia* sequences.
